# Antiacetylcholinesterase triterpenes from the fruits of *Cimicifuga yunnanensis*†

**DOI:** 10.1039/c8ra00291f

**Published:** 2018-02-19

**Authors:** Yin Nian, Ni-Hong Lu, Xiao-Ling Liu, Da-Shan Li, Lin Zhou, Ming-Hua Qiu

**Affiliations:** State Key Laboratory of Phytochemistry and Plant Resources in West China, Kunming Institute of Botany, Chinese Academy of Sciences Kunming 650201 People's Republic of China mhchiu@mail.kib.ac.cn; Department of Respiratory Medicine, The Third People's Hospital of Kunming Kunming 650041 People's Republic of China

## Abstract

Two new cycloartane triterpenes, cimyunnin E (1), containing a unique oxaspiro[4.4]nonanedione moiety based on rings D and E, together with cimicifine B (2), a 25,26,27-trinortriterpene featuring a pyridine ring E, were purified from the fruits of *Cimicifuga yunnanensis*. Their structures were elucidated by spectroscopic methods and ECD (electronic circular dichroism calculations). Compounds 1 and 2 showed significant acetylcholinesterase (AChE) inhibition with IC_50_ values of 1.58 and 3.87 μM, respectively. In addition, they noticeably enhanced the neurite outgrowth of nerve growth factor (NGF) mediated PC12 cells at a concentration of 10 μM.

## Abstract 2

Two novel cycloartane triterpenes, cimyunnin E (1) and cimicifine B (2), were purified from the fruits of *Cimicifuga yunnanensis*. Significantly, these two compounds showed potent *anti*-AChE effects and neurotrophic activities.

## Graphical abstract

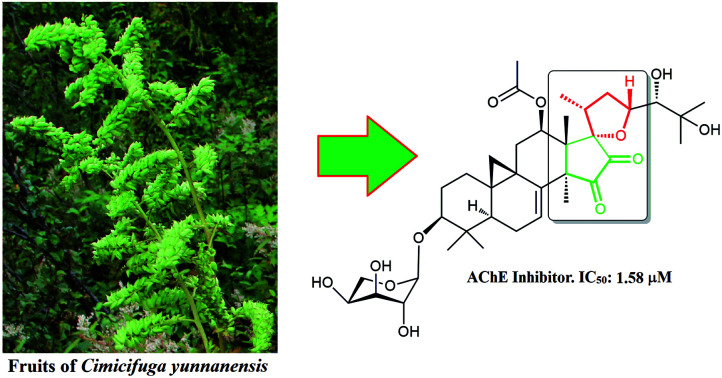

## Introduction

Plants of the *Cimicifuga* genus, such as *C. racemosa*, *C. foetida*, *C. dahurica*, *C. heracleifolia*, *C. simplex*, and *C. japonica*, are well-known herbal medicines in Europe, the United States, and East Asia.^1–4^ In the past few decades, more than 300 9,19-cycloartane triterpenes (CTs), which are considered to be the main active chemical constituents of this genus, have been reported.^5–30^ Moreover, biological evaluations revealed these CTs possessed a variety of activities, such as cytotoxicity,^5,12–14^ antiosteoporotic,^31^ anti-AIDS,^32^ immunosuppression,^33^ anti-angiogenic,^6^ and anti-Alzheimer.^34^

**Fig. 1:**
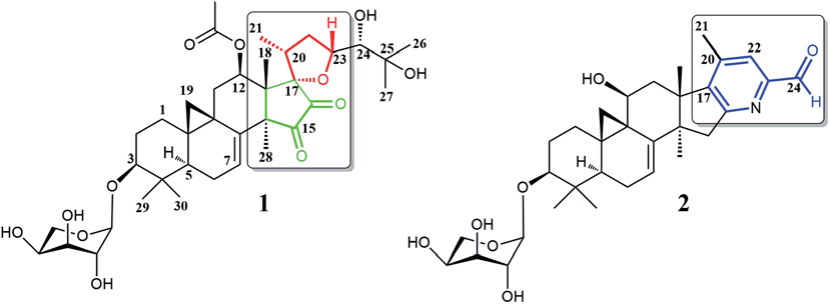
Structures of compounds 1 and 2.

It is worth mentioning that previous studies had mainly focused on the roots of aforementioned *Cimicifuga* spp.^5–30^ In an attempt to further explore structurally and biologically interesting CTs from this genus, we started to study some rare species, such as *C. yunnanensis* and *C. frigida*, particularly, on the fruits and flowers of these plants. As a result, a number of novel bioactive CTs were discovered. Cimyunnin A, a CTs possessed an unique cyclopentenone ring G from the fruits of *C. yunnanensis*, was considered as an anti-angiogenic leading structure.^6^ In addition, cimifrigines A–G, a series of cytotoxic CTs featured by an unusual oxime group at C-15, were identified from the flowers of *C. frigida*.^35^ Be motivated by these findings, we continually carried out phytochemical and pharmaceutical studies on the fruits of *C. yunnanensis*. Consequently, another two novel triterpenes, cimyunnin E (1) and cimicifine B (2), were isolated and identified (Fig. 1). Compound 1 represents the first example of CTs with an unprecedented oxaspiro[4.4]nonanedione unite formed in ring D and E. While, compounds 2 is a trinortriterpene containing a pyridine ring E. Significantly, biological evaluations revealed that compounds 1 and 2 were strong AChE inhibitors with IC_50_ values of 1.58 and 3.87 μM, respectively (the IC_50_ value of positive control tacrine is 0.17 μM, see Table S1 and Fig. S22†). Moreover, the neuronal differentiation of NGF-mediated PC12 cells were also enhanced by compounds 1 and 2 at the concentration of 10 μM (Table S2 and Fig. S23†). Described herein are the isolation, structure elucidation, and biological activities of these compounds.

## Results and discussion

Cimyunnin E (1), white powder, had the molecular formula C_37_H_54_O_12_ as determined by the HRESIMS at *m*/*z* 713.3515 [M + Na]^+^ (calcd 713.3513), corresponding to 11 double-bond equivalents. The IR spectrum showed absorptions attributable to OH (3425 cm^−1^), carbonyl (1745 cm^−1^) and olefinic (1603 cm^−1^) groups, respectively. In the ^1^H NMR spectrum (Table 1), downfield shifted cyclopropane methylene signals at *δ*_H_ 0.69 and 1.04 (each 1H, d, *J* = 3.6 Hz) and an anomeric proton resonance at *δ*_H_ 4.76 (d, *J* = 7.1 Hz) were observed. The ^13^C (Table 1) and HMQC spectra revealed the existence of two olefinic carbons at *δ*_C_ 117.0 (C-7, d) and 140.5 (C-8, s), an ester carbonyl group at *δ*_C_ 170.1 (s), two carbonyl carbons at *δ*_C_ 199.9 (C-15, s) and 204.6 (C-16, s), and six oxygenated carbon atoms at *δ*_C_ 87.6 (C-3, d), 67.2 (C-12, d), 87.4 (C-17, s), 78.3 (C-23, d), 79.4 (C-24, d), and 72.1 (C-25, s), respectively. These data suggested that 1 was a highly oxygenated CTs glycoside with a six-ring skeleton.

**Table 1:** ^1^H and ^13^C NMR data of compounds 1 and 2 (*δ* in ppm, *J* in Hz)

Position	1		2	
	*δ*_H_ b	*δ*_C_ c	*δ*_H_ b	*δ*_C_ c
1	1.57^a^, 1.23 m	29.9 t	2.76 m, 1.70 m	27.2 t
2	2.29 m, 1.88 m	29.2 t	2.42 brd (11.8), 2.13 brd (12.8)	29.6 t
3	3.44 dd (11.5, 3.7)	87.6 d	3.61 dd (11.5, 4.1)	88.2 d
4		40.2 s		40.6 s
5	1.18 m	41.4 d	1.38^a^	43.7 d
6	1.94 m, 1.53 m	21.5 t	2.00 m, 1.81 m	21.9 t
7	6.60 d (6.9)	117.0 d	5.36 d (7.0)	115.2 d
8		140.5 s		145.8 s
9		20.7 s		27.5 s
10		28.0 s		29.3 s
11	2.76 dd (15.8, 7.8), 1.42 brd (16.3)	35.3 t	4.62 m	62.8 d
12	6.20 d (7.6)	67.2 d	3.08 dd (13.4, 9.5), 2.47 brd (11.3)	43.8 t
13		46.8 s		49.2 s
14		54.9 s		51.1 s
15		199.9 s	3.32 d (15.4), 3.01 d (15.4)	43.6 t
16		204.6 s		165.2 s
17		87.4 s		148.4 s
18	1.37 s	18.5 q	1.38 s	23.7 q
19	1.04 d (3.6), 0.69 d (3.6)	26.8 t	2.04 d (3.7), 1.08^a^	18.7 t
20	2.55 m	37.0 d		142.8 s
21	1.29 brd (8.3)	18.6 q	2.22 s	17.9 q
22	2.92 dd (20.2, 8.3), 1.93 m	38.7 t	7.66 s	123.1 d
23	4.54 m	78.3 d		151.4 s
24	3.74 brd (6.3)	79.4 d	10.22 s	193.7 s
25		72.1 s		
26	1.58 s	28.1 q		
27	1.50 s	25.8 q		
28	1.67 s	27.0 q	1.06 s	28.3 q
29	1.30 s	25.5 q	1.42 s	25.8 q
30	1.00 s	13.9 q	1.15 s	14.3 q
3-Ara				
1′	4.76 d (7.1)	107.3 d	4.84 d (7.0)	107.3 d
2′	4.45 t (7.9)	72.8 d	4.47 m	72.8 d
3′	4.16 dd (8.9, 2.6)	74.5 d	4.18 m	74.5 d
4′	4.30 brs	69.5 d	4.31^a^	69.4 d
5′	4.28 brd (12.6), 3.78 brd (12.6)	66.8 t	4.30 m, 3.79 m	66.7 t
12-OCOCH_3_	2.12 s			
12-OCOCH_3_		21.4 q		
12-OCOCH_3_		170.1 s		

a Signals overlapped.

b Recorded at 600 MHz in pyridine-*d*_5_.

c Recorded at 150 MHz in pyridine-*d*_5_.

**Fig. 2:**
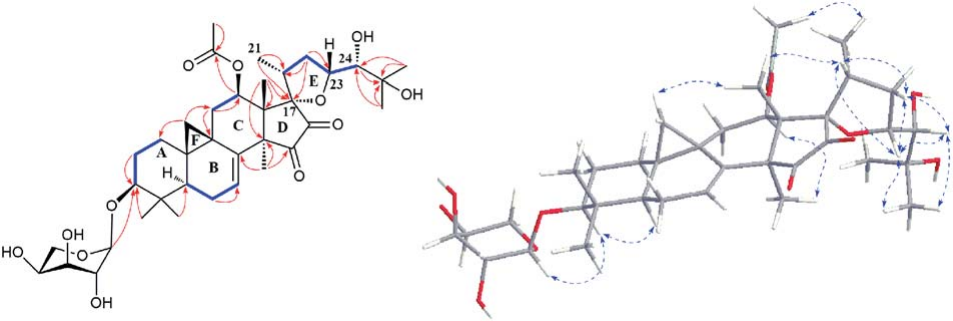
Major HMBC (
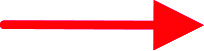
), ^1^H–^1^H COSY (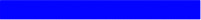), and ROESY (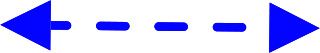) correlations of compound 1.

The detailed 1D and 2D NMR analyses established the planar structure of 1. The ^1^H–^1^H COSY (Fig. 2) spectrum disclosed that 1 has partial structures –CH_2_CH_2_CH– (due to C-1 to C-3), –CHCH_2_CH– (for C-5 to C-7), and –CH_2_CH– (for C-11 to C-12), that were compatible for rings A, B, and C of the CTs with a pair of double bond at C-7 and C-8.^27,29^ The existence of the downfield shifted cyclopropane methylene (*δ*_H_ 0.69 and 1.04) further supported this deduction. The acetoxy group was located to C-12 on the basis of the HMBC correlation of H-12 (*δ*_H_ 6.20) and the ester carbonyl group (*δ*_C_ 170.1). Similarly, the sugar unit was attached to C-3 by the HMBC coupling between the anomeric proton (*δ*_H_ 4.76) and C-3 (*δ*_C_ 87.6). In addition, the sugar obtained after acid hydrolysis was identified as l-arabinose by comparing its TLC and specific rotation with a standard. Thus, ring A, B, C, F, and the sugar unit of 1 was constructed as shown (Fig. 2).

The spin system –CH_3_CHCH_2_CHCH– due to Me-21, C-20, C-22, C-23 and C-24 was also deduced from ^1^H–^1^H COSY correlations (Fig. 2). In addition, HMBC correlations from CH_3_-21 (*δ*_H_ 1.29) and H-22 (2.92, 1H) to the oxygenated quaternary carbon at *δ*_C_ 87.4 (C-17) indicated the linkage of C-20 and C-17 (Fig. 2). Similarly, the connection of C-24 to the isopropanol group (C-25, C-26, and C-27) was elucidated by the HMBC couplings of H-24 (*δ*_H_ 3.74) CH_3_-26 (*δ*_H_ 1.58) and CH_3_-27 (*δ*_H_ 1.50) to the hydroxyl substituted quaternary carbon at *δ*_C_ 72.1 (C-25). By now, there were still one oxygen atom and two carbonyl carbons (C-15, *δ*_C_ 199.9; C-16, *δ*_C_ 204.6) need to be assigned and four degree of unsaturation unaccounted for, requiring another two rings in the final structure. In the HMBC spectrum, correlations of CH_3_-28 (*δ*_H_ 1.67) to C-14 (*δ*_C_ 54.9) and C-15 (*δ*_C_ 199.9) were observed, indicating the connection between C-14 and C-15. Similarly, the connection of C-13 and C-17 was deduced from HMBC correlations of CH_3_-18 (*δ*_H_ 1.37) to C-13 (*δ*_C_ 46.8), and C-17 (*δ*_C_ 87.4), respectively. Thus, to fulfill the unsaturation requirement, the molecular weight, and the chemical shift of C-15 and C-16, C-17 was connected to C-16 and C-23 was linked to C-17 by an oxygen atom (based on HMBC correlations, C-17 and C-23 could be connected by the carbonyl carbon C-16. Thus, ring D would be a five-membered lactone ring. In that case, the chemical shift for C-15 should around *δ*_C_ 170.0). Therefore, an unique 15,16-cyclopentanedione-17-spiro-17,23-oxolane moiety was established in 1. Finally, the planar structure of 1 was constructed as shown (Fig. 2).

**Fig. 3:**
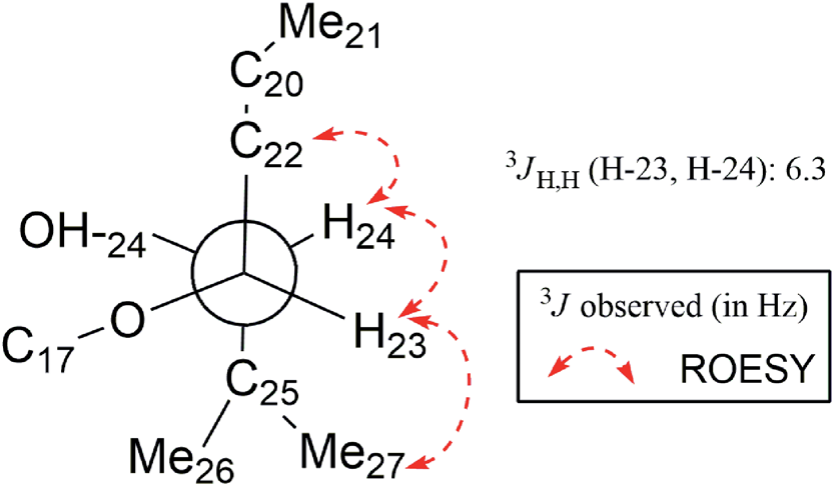
The Newman projection of C-23/C-24 coupling system of 1.

**Fig. 4:**
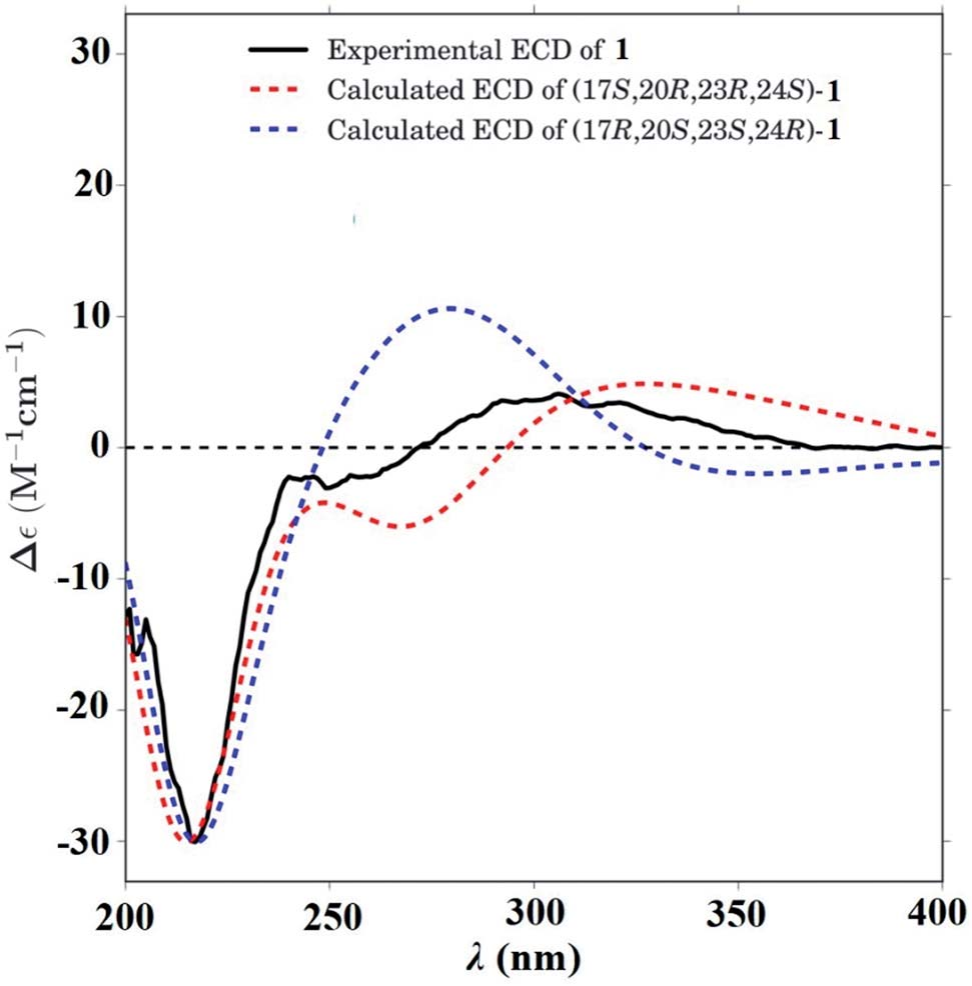
Calculated ECD spectra for -(17*S*,20*R*,23*R*,24*S*)-1 (red dashed line) and -(17*R*,20*S*,23*S*,24*R*)-1 (blue dashed line). Experimental CD spectrum of 1 (black solid line).

In the ROESY spectrum (Fig. 2), correlations of H-5 (biogenetically α-oriented)/H-3, H-3/H-1′, and Me-28 (biogenetically α-oriented)/H-12 indicated the α-orientation of H-1′, H-3, and H-12. Our efforts to make fine crystals from 1 failed which precluded the possibility to determine the absolute configuration directly by X-ray crystallography. However, the diagnostic ROESY couplings of Me-18 (biogenetically β-oriented) to H-20, H-20 to H-23, and acetoxy methyl to Me-21 (biogenetically α-oriented) were observed, which help to determine β-orientation of H-20 and H-23 and the conformation of ring E as shown (Fig. 2). Therefore, the relative configuration of C-17, C-20, and C-23 of 1 was assigned as S*, R*, and R*, respectively. For the 17R*, 20S*, and 23S* stereoisomer of 1, those correlations would not be observed (except H-20 to H-23, Fig. S21†), which further confirmed this deduction. On the basis of ROESY correlations of H-23/H-24, H-24/H-22, and H-23/Me-27 and the ^3^*J*_H,H_ value of H-23 and H-24 (6.3 Hz), the Newman projection of C-23/C-24 coupling system was established as shown (Fig. 3), which help to establish the S* configuration of C-24. Finally, the ECD calculation was applied to determine the absolute configuration of 1. As shown in Fig. 4, spectrum calculated for the 17*S*, 20*R*, 23*R*, and 24*S* one was nearly identical with the experimental data of 1 over the whole range of wavelengths under investigation, whereas the stereoisomer exhibited very different ECD behaviour between 250–300 nm. Therefore, the absolute configurations of C-17, C-20, C-23, and C-24 of 1 were determined as *S*, *R*, *R*, and *S*, respectively.

The molecular composition of cimicifine B (2), C_32_H_43_NO_7_, was deduced from HRESIMS ([M + Na]^+^, *m*/*z* 576.2931), indicating 12 degrees of unsaturation. In the ^1^H NMR spectrum (Table 1) of 2, signals due to an extremely downfield shifted cyclopropane methylene at *δ*_H_ 1.08 (overlapped) and 2.04 (d, *J* = 3.7 Hz), an anomeric proton at *δ*_H_ 4.84 (d, *J* = 7.0 Hz), two olefinic hydrogen atoms at *δ*_H_ 5.37 (d, *J* = 7.0 Hz) and 7.66 (s), five tertiary methyl groups at *δ*_H_ 1.06–1.42, and an active hydrogen signal at *δ*_H_ 10.22, were observed. The ^13^C NMR and DEPT (Table 1) spectra displayed signals for a carbonyl group (*δ*_C_ 193.7, C-24), a hydroxylmethine (*δ*_C_ 62.8, C-11), two trisubstituted olefinic carbons (*δ*_C_ 115.2, C-7; *δ*_C_ 123.1, C-22), and five tetrasubstituted olefinic groups (*δ*_C_ 145.8, C-8; *δ*_C_ 165.2, C-16; *δ*_C_ 148.4, C-17; *δ*_C_ 142.8, C-20; *δ*_C_ 151.4, C-23). Therefore, aforementioned data suggested that 2 was a trinortriterpene glycoside and a six-ring structure, which included an unsaturated azacycle, was required to fulfill the unsaturation requirement.

**Fig. 5:**
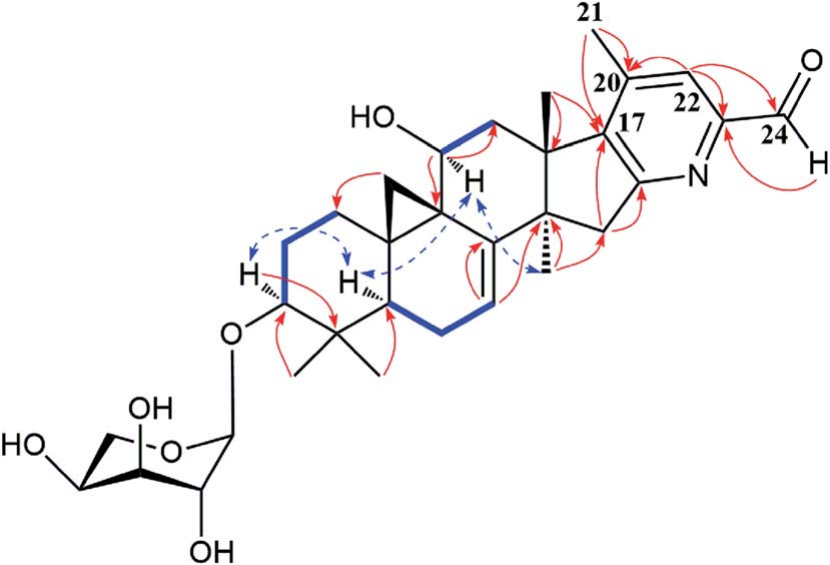
Major HMBC (
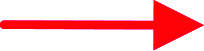
), ^1^H–^1^H COSY (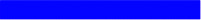), and ROESY (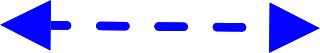) correlations of compound 2.

Extensive analyses of 2D NMR spectra revealed the conformation of ring A, B, C, D, and F of 2 as shown (Fig. 5), which was similar to those of known compounds.^5–30^ In the ^1^H–^1^H COSY spectrum (Fig. 5), the correlations of H-5 (*δ*_H_ 1.38) to H-6 (*δ*_H_ 1.81 and 2.00, each 1H) and H-6 to the olefinic proton (*δ*_H_ 5.37, H-7) indicated a pair of double bond at C-7 and C-8. The ^1^H–^1^H COSY association of the proton (*δ*_H_ 4.62) of hydroxymethine (*δ*_C_ 62.8, C-11) to H-12 (*δ*_H_ 2.47 and 3.08, each 1H), together with the extremely downfield shifted proton signals of C-19 determined the location of a hydroxyl group at C-11. In the HMBC spectrum (Fig. 5), the couplings of H-15 (*δ*_H_ 3.01 and 3.32, each 1H) to C-16 (*δ*_C_ 165.2) and C-17 (*δ*_C_ 148.4), and Me-18 (*δ*_H_ 1.38) to C-17 (*δ*_C_ 148.4) indicated the double bond between C-16 and C-17. Further analysis of HMBC spectrum revealed the correlations of Me-21 (*δ*_H_ 2.22) to C-20 (*δ*_C_ 142.8) and C-17 (*δ*_C_ 148.4), and H-22 (*δ*_H_ 7.66) to C-20 (*δ*_C_ 142.8) and C-23 (*δ*_C_ 151.4), indicating the linkage of C-17/C-20/C-22/C-23. In addition, the connection between C-23 and C-24 was elucidated by the HMBC correlation of H-22 (*δ*_H_ 7.66) to the carbonyl carbon (*δ*_C_ 193.7, C-24). Thus, to fulfill the double-bond equivalents, a pyridine ring E should be fused to ring D through C-16 and C-17. Similarly, to fulfill the molecular weight of 2 an aldehyde group was assigned to C-24, which was further supported by the HMBC correlation of an active hydrogen (*δ*_H_ 10.22) and C-23 (*δ*_C_ 151.4). Finally, by the same way as that of 1, the sugar was identified as l-arabinose and located at C-3. The orientations of H-3 and H-11 were ascribed by the ROESY correlations (Fig. 5) between H-5 (biogenetically α-oriented) and H-3, and H-11. Thus, the structure of 2 was determined as shown.

Hypothetically, prevention of acetylcholine (Ach) hydrolysis could increase the efficiency of cholinergic transmissions, which has been reported to be associated with the onset of Alzheimer's disease (AD).^36^ Thus, enhancement of ACh levels by potent AChE inhibitors in the brain has been considered to be an effective approach for treating AD.^37–39^ Although prominent biological activities of CTs from *Cimicifuga* spp. have been reported, to date, however, *anti*-AChE knowledge of those chemical constituents is mainly not yet involved. Thus, the AChE inhibitory activities of compounds 1 and 2 were evaluated using the Ellman method.^40^ Unexpectedly, compound 1 exhibited significant inhibition on AChE with an IC_50_ value of 1.58 μM (Table S1 and Fig. S22†). Similarly, compound 2 showed noticeable inhibitory effect on AChE, having an IC_50_ value of 3.87 μM. Tacrine was used as the positive control and had an IC_50_ value of 0.17 μM.

AD is a type of neurodegenerative diseases. Any agent with neurotrophic activity may benefit AD. Therefore, the effects of 1 and 2 to stimulate NGF-mediated neurite outgrowth on PC12 cells were further evaluated. As a result, 1 and 2 obviously increased the neuronal differentiation at a concentration of 10 μM. The differentiation rates are 15.34% and 11.72% for 1 and 2, respectively, compared with 4.17% of the negative control and 19.18% of the positive control (Table S2 and Fig. S23†).

## Conclusions

In our continual investigation on the fruits of *C. yunnanensis*, another two unusual CTs were obtained. Cimyunnin E (1) is the first CTs possessing an oxaspiro[4.4]nonanedione moiety in ring D and E. While, cimicifine B (2) is a trinortriterpene contains a fused pyridine ring E. Significantly, these two compounds showed potent *anti*-AChE effects and neurotrophic activities. Therefore, the bioactivities of cimyunnin E and cimicifine B deserve further study. In summary, once again, novel active constituents were isolated from fruits of *C. yunnanensis*, the more sophisticated parts of this genus, such as pollen and vegetative organ are worth studying in future.

## Experimental section

### General experimental procedures

A JASCO P-1020 digital polarimeter was applied to record optical rotations, using MeOH as solvent. 1D and 2D NMR spectra were performed on Bruker DRX-500 and Avance III-600 MHz spectrometers (Bruker, Zűrich, Switzerland) with solvent signal as internal reference. ESIMS and HRESIMS were run on an Agilent G6230 TOF MS (Agilent Technologies, Palo Alto, USA). Infrared spectra were tested on a Shimadzu IR-450 instrument with KBr pellets. Column chromatography (CC) was run on Silica gel (200–300 mesh, Qingdao Marine Chemical, Inc.), and Lichroprep RP-18 (40–63 μm, Merck). Semipreparative HPLC was carried out on an Agilent 1100 liquid chromatography system using an YMC-Pack 10 mm × 250 mm column (Pro C18 RS). Precoated TLC plates (200–250 μm thickness, silica gel 60 F_254_, Qingdao Marine Chemical, Inc.) were used for thin-layer chromatography. The spots in TLC were visualized by heating after spraying with 10% aq. H_2_SO_4_.

### Plant material

The fruits of *Cimicifuga yunnanensis* (1.5 kg) were collected from Bomi County, Tibet, China, in September 2013. Prof. Wang Zongyu, Kunming Institute of Botany, Chinese Academy of Sciences, identified the species. A voucher specimen (KUN no. 201309007) has been deposited at the State Key Laboratory of Phytochemistry and Plant Resources in West China, Kunming Institute of Botany, Chinese Academy of Sciences, PR China.

### Extraction and isolation

MeOH (6 L, 3 times, 7 days each) was used to extract the dried and milled fruits of *Cimicifuga yunnanensis* (1.5 kg) at room temperature. MeOH was evaporated under vacuum at 50 °C to afford the extract (156.3 g). The extract gave fractions A (28.2 g), B (31.3 g), C (29.5 g), D (27.3 g) and E (12.7 g) by silica gel CC (3.0 kg, 10 × 150 cm) eluted with CHCl_3_–MeOH [100 : 0 (6 L), 50 : 1 (10 L), 10 : 1 (10 L), 5 : 1 (7 L), 0 : 100 (4 L)]. Subsequently, five sub-fractions (C.1–C.5) were obtained through RP-18 CC (1000 g, 12 × 60 cm), gradiently eluted with MeOH–H_2_O from 0 : 100 to 100 : 0. Fractions (C.3.1–C.3.4) were obtained by further RP-18 CC (eluted with MeOH–H_2_O, gradient from 40 : 60 to 85 : 15) on fraction C.3. Compound 1 (3.5 mg) was purified from fraction C.3.2 (1.4 g) by silica gel CC (40 g, 3 × 40 cm) eluted with CHCl_3_–Me_2_CO (gradient from 10 : 1 to 5 : 1), and then repeated semipreparative HPLC (eluted with CH_3_CN–H_2_O, gradient from 50 : 50 to 65 : 35). Fraction C.2 (3.9 g) yielded compound 2 (2.6 mg) by silica gel CC (40 g, 3 × 60 cm) eluting with CHCl_3_–Me_2_CO from 20 : 1 gradient to 10 : 1 and repeated semipreparative HPLC (eluted with CH_3_CN–H_2_O, gradient from 55 : 45 to 70 : 30).

#### (3β,12β,17*S*,20*R*,23*R*,24*S*)-17,23-Epoxy-15,16-dione-12-acetoxy-3,25-dihydroxy-7-en-9,19-cycloart-3-*O*-α-larabinopyranoside (1)

White powder; [*α*]^24^_D_ = −304.0 (*c* 0.10, MeOH); IR (KBr): *ν*_max_ 3425, 2927, 2853, 1745, 1603, 1460, 1382, 1242, 1085, 989 cm^−1^; UV (MeOH) *λ*_max_ (log *ε*): 205 (0.28), 250 (0.04); see Table 1 for ^1^H NMR (600 MHz, C_5_D_5_N) and ^13^C NMR (150 MHz, C_5_D_5_N) data; positive ESIMS [M + Na]^+^*m*/*z* 713; positive HRESIMS [M + Na]^+^*m*/*z* 713.3515 (calcd for C_37_H_54_NaO_12_, 713.3513).

#### (3β,11β)-11-Hydroxy-16,23-nitrilo-7,17(20),22-trien-24-formyl-25,26,27-trinor-9,19-cycloart-3-*O*-α-larabinopyranoside (2)

White powder; [*α*]^19^_D_ = −4.8 (*c* 0.13, MeOH); IR (KBr): *ν*_max_ 3426, 2927, 2854, 1707, 1630, 1599, 1451, 1383, 1259, 1070, 589 cm^−1^; UV (MeOH) *λ*_max_ (log *ε*): 203 (0.55), 273 (0.13); see Table 1 for ^1^H NMR (600 MHz, C_5_D_5_N) and ^13^C NMR (150 MHz, C_5_D_5_N) data; positive ESIMS [M + Na]^+^*m*/*z* 576; positive HRESIMS [M + Na]^+^*m*/*z* 576.2931 (calcd for C_32_H_43_NNaO_7_, 576.2937).

### Hydrolysis and identification of the sugar units in compounds 1 and 2

The MeOH solution (3 mL) of each compound (1.5 mg) was refluxed with 0.5 N HCl (2 mL) for 2 h. CHCl_3_ (3 × 6 mL) was used to extract the reaction mixture after diluting with H_2_O. A monosaccharide was given by neutralizing each aqueous layer with Ag_2_CO_3_ and filtering the precipitate. The monosaccharide from compounds 1 and 2 had an *R*_f_ (EtOAc–CHCl_3_–MeOH–H_2_O, 3 : 2 : 2 : 1) and specific rotation of [*α*]^20^_D_ +85.4 (*c* 0.08, MeOH) corresponding to those of l-arabinose (Sigma-Aldrich).

### Acetylcholinesterase inhibitory activity

Acetylcholinesterase (AChE) inhibitory activities of compounds 1 and 2 were assayed by the spectrophotometric method developed by Ellman *et al.*^40^ with slightly modification. *S*-Acetylthiocholine iodide, 5,5′-dithio-bis-(2-nitrobenzoic) acid (DTNB, Ellman's reagent), and acetylcholinesterase derived from human erythrocytes were purchased from Sigma Chemical. Compounds 1 and 2 were dissolved in DMSO. The reaction mixture (totally 200 μL) containing phosphate buffer (pH 8.0), test compound (50 μM for preliminary screening; 100, 50, 30, 10, 3, 1, and 0.2 μM for IC_50_ value assay), and acetylcholinesterase (0.02 U mL^−1^) was incubated for 20 min (30 °C). Then, the reaction was initiated by the addition of 40 μL of solution containing DTNB (0.625 mM) and acetylthiocholine iodide (0.625 mM) for AChE inhibitory activity assay. The hydrolysis of acetylthiocholine was monitored at 405 nm every 30 seconds for one hour. Tacrine was used as positive control with final concentration of 0.333 μM for preliminary screening and 2, 1, 0.5, 0.2, 0.04, 0.008, and 0.0016 μM for IC_50_ value assay. All the reactions were performed in triplicate. The percentage inhibition was calculated as follows: % inhibition = (NC − *S*)/NC × 100 (NC (negative control) is the activity of the enzyme without test compound and with 2% DMSO and *S* is the activity of enzyme with test compound and the final concentration of DMSO is 0.1%). Inhibition curves were obtained for each compound by plotting the percent inhibition *versus* the logarithm of inhibitor concentration in the assay solution. The linear regression parameters were determined for each curve and the IC_50_ values extrapolated. The same procedure was applied for the positive control tacrine.

### Neurite outgrowth-promoting activity

The neurotrophic activities of the tested compounds were examined according to an assay using PC12 cells as reported.^41^ Briefly, PC12 cells (purchased from Kunming institute of zoology) were maintained in F12 medium (Ham's F12K, Gibco's reagent) supplemented with 12.5% horse serum (HS, Hyclone's reagent), and 2.5% fetal bovine serum (FBS, Hyclone's reagent), and incubated at 5% CO_2_ and 37 °C. Tested compounds were dissolved in DMSO. For the neurite outgrowth-promoting activity bioassay, PC12 cells were seeded at a density of 5 × 10^4^ cells per mL in 48-well plate coated with poly-l-lysine (sigma's reagent). After 24 h, the medium was changed to that containing 10 μM of each test compounds plus 5 ng mL^−1^ NGF (sigma's reagent), or different concentrations of NGF (50 ng mL^−1^ for the positive control, 5 ng mL^−1^ for the negative control). The final concentration of DMSO was 0.05%, and the same concentration of DMSO was added into the negative control. After 72 h incubation, the neurite outgrowth was assessed under a phase contrast microscope. Neurite processes with a length equal to or greater than the diameter of the neuron cell body were scored as neurite bearing cells. The ratio of the neurite-bearing cells to total cells (with at least 100 cells examined per view area; 5 viewing area per well) was determined and expressed as a percentage.

### ECD calculation

The theoretical calculations were carried out using Gaussian 09.^42^ Structures were first optimized at PM6 using semi-empirical theory method and then optimized at HF/6-31G(d) theory level. Room-temperature equilibrium populations were calculated according to Boltzmann distribution law (eqn. (1)). The conformers with Boltzmann-population of over 1% were chosen and further optimized at B3LYP/6-311G(d,p) in methanol using the IEFPCM model (Table S3†). Vibrational frequency analysis confirmed the stable structures.1
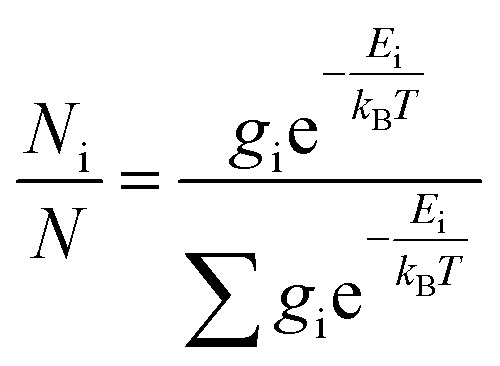
where *N*_i_ is the number of conformer i with energy *E*_i_ and degeneracy *g*_i_ at temperature *T*, and *k*_B_ is Boltzmann constant. Under the same condition, the ECD calculation was conducted using time-dependent density functional theory (TD-DFT). Rotatory strengths for a total of 30 excited states were calculated. The ECD spectrum was simulated in SpecDis^43^ by overlapping Gaussian functions for each transition according to (eqn. (2)):2
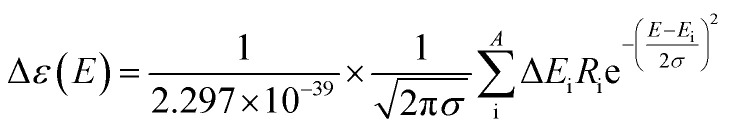
where *σ* represents the width of the band at 1/*e* height, and Δ*E*_i_ and *R*_i_ are the excitation energies and rotatory strengths for transition i, respectively. Parameters of *σ* and UV-shift for enantiomers were 0.5 eV and 1 nm, respectively.

## Conflicts of interest

There are no conflicts of interest to declare.

## Supplementary Material

RA-008-C8RA00291F-s001
