# Supplementary material for: Antiacetylcholinesterase triterpenes from the fruits of Cimicifuga yunnanensis
Source: RSC Adv. 2018 Feb 19;8(14):7832–8. doi: 10.1039/c8ra00291f (PMC9078504; doi:10.1039/c8ra00291f)
Supplement: RA-008-C8RA00291F-s001 [file RA-008-C8RA00291F-s001.pdf]

Electronic Supplementary Material (ESI) for RSC Advances.
This journal is © The Royal Society of Chemistry 2018

Antiacetylcholinesterase Triterpenes from the Fruits of *Cimicifuga yunnanensis*

Yin Nian,[a,‡] Ni-Hong Lu,[b,‡] Xiao-Ling Liu,[a,‡] Da-Shan Li,[a] Lin Zhou,[a] Ming-Hua Qiu[*,a]

[a]*State Key Laboratory of Phytochemistry and Plant Resources in West China, Institute of Botany, Chinese Academy of Sciences, Kunming 650201, People's of China*

[b]*Department of Respiratory Medicine, the Third People's Hospital of Kunming, Kunming 650041, People's Republic of China*

Supporting Information List

Page 3-13, ^{1}H, ^{13}C, HSQC, HMBC, COSY and ROESY NMR spectra, UV, IR, and CD spectra, and OR and HREIMS experiments of compound **1**.

Page 14-23, ^{1}H, ^{13}C, HSQC, HMBC, COSY and ROESY NMR spectra, UV and IR spectra, and OR and HREIMS experiments of compound **2**.

Page 24, **Figure S21.** Structure of 17*R*, 20*S*, 23*S*, and 24*R* stereoisomer of **1** and unlikely happened ROESY correlations.

Page 25, **Table S1.** Dose-related inhibitions of compounds **1** and **2** on acetylcholinesterase (AChE).

Page 26, **Figure S22**. Dose-response relationships of compounds **1** (A), **2** (B), and Tacrine (C).

Page 27, **Table S2.** The effects of compounds **1** and **2** on the NGF mediated neurite outgrowth of PC12 cells

Page 28, **Figure S23.** Represented pictures of the NGF mediated neurite outgrowth of PC12 cells.

Page 29-54, Computational methods of **1.**

Figure S1. ^{1}H (600 Hz) NMR Spectrum of cimyunnin E (**1**) in Pyridine-d_5

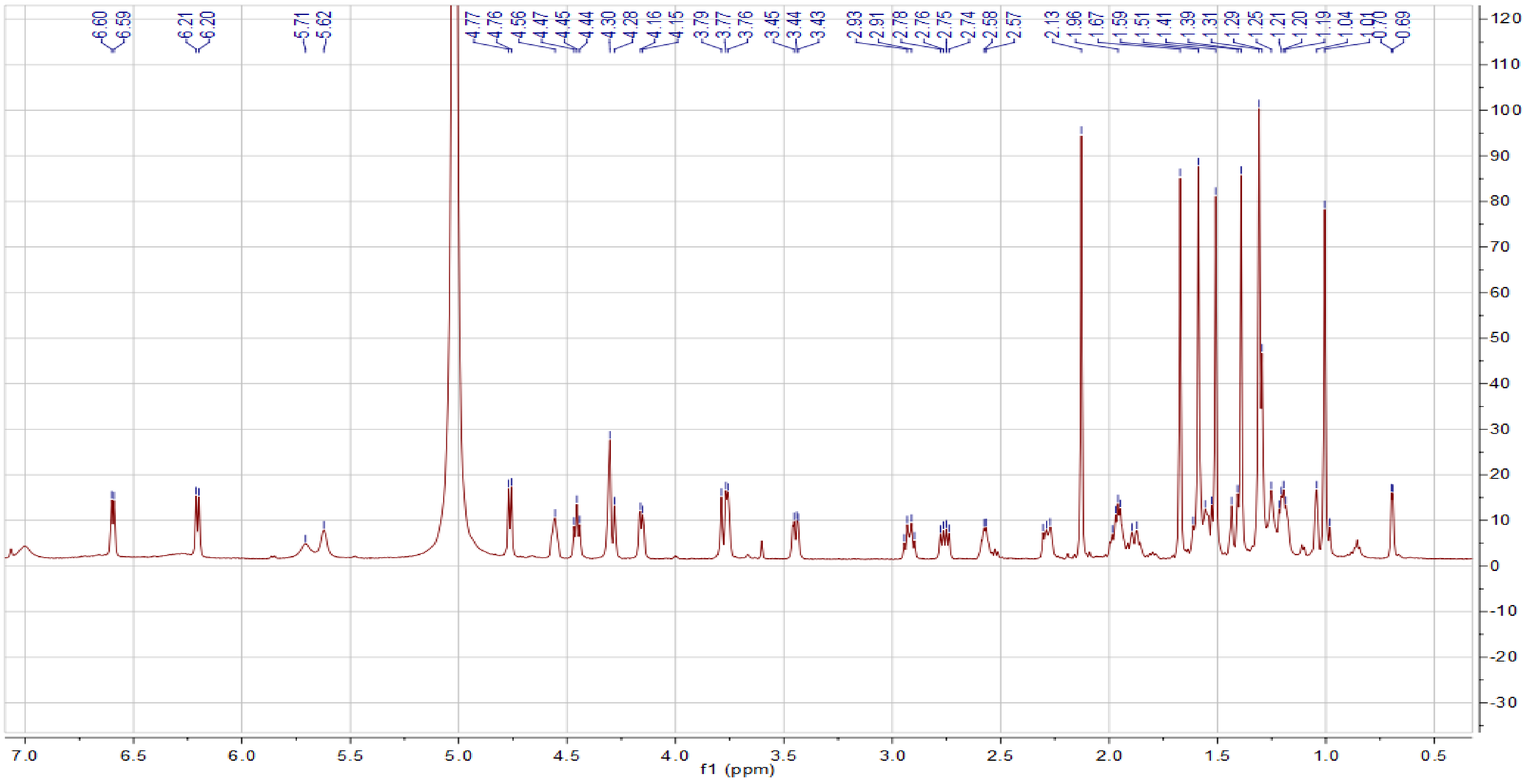

Figure S2. ^{13}C (150 Hz) NMR Spectrum of cimyunnin E (**1**) in Pyridine-d_5

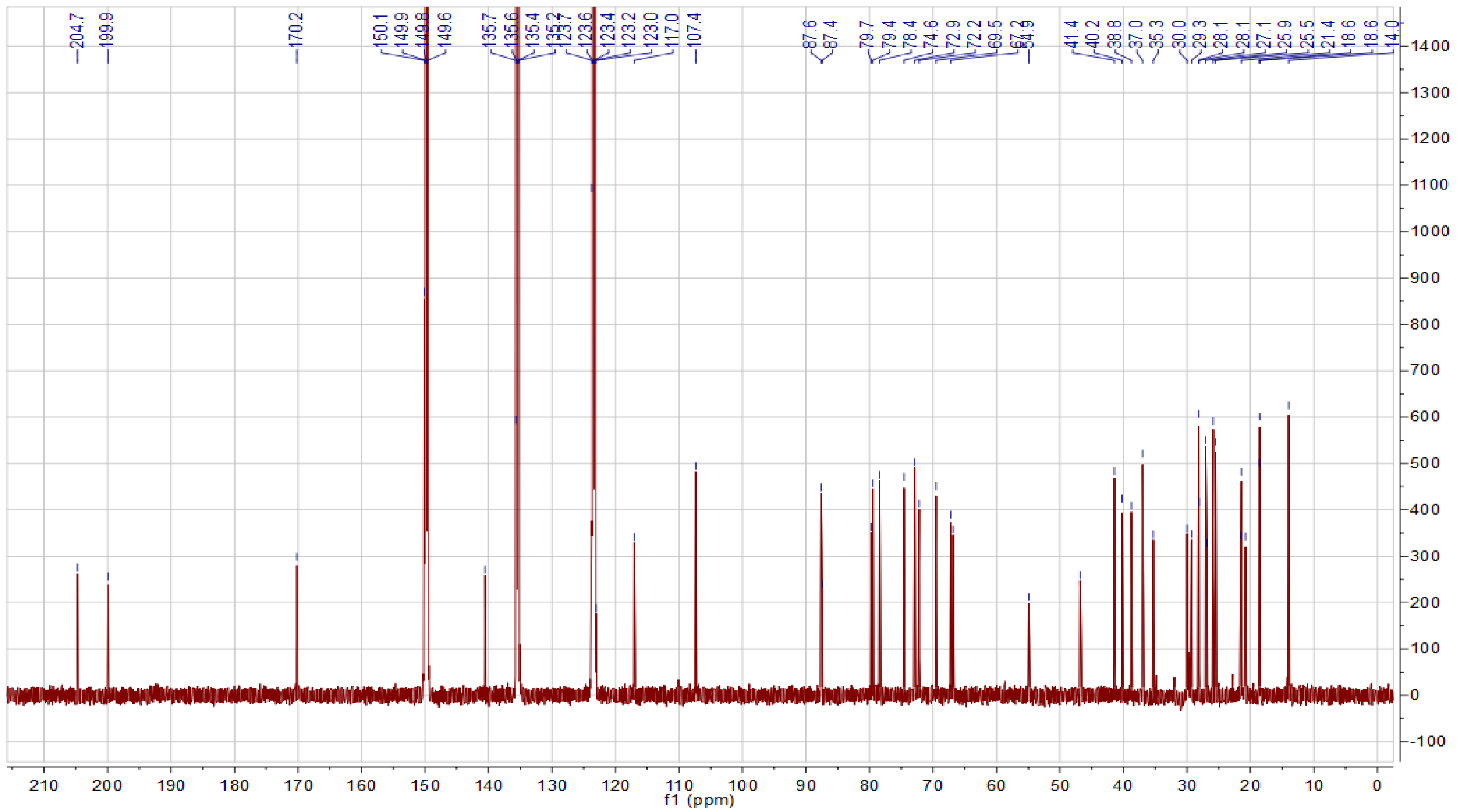

Figure S3. HSQC (600 Hz) Spectrum of cimyunnin E (**1**) in Pyridine-d_5

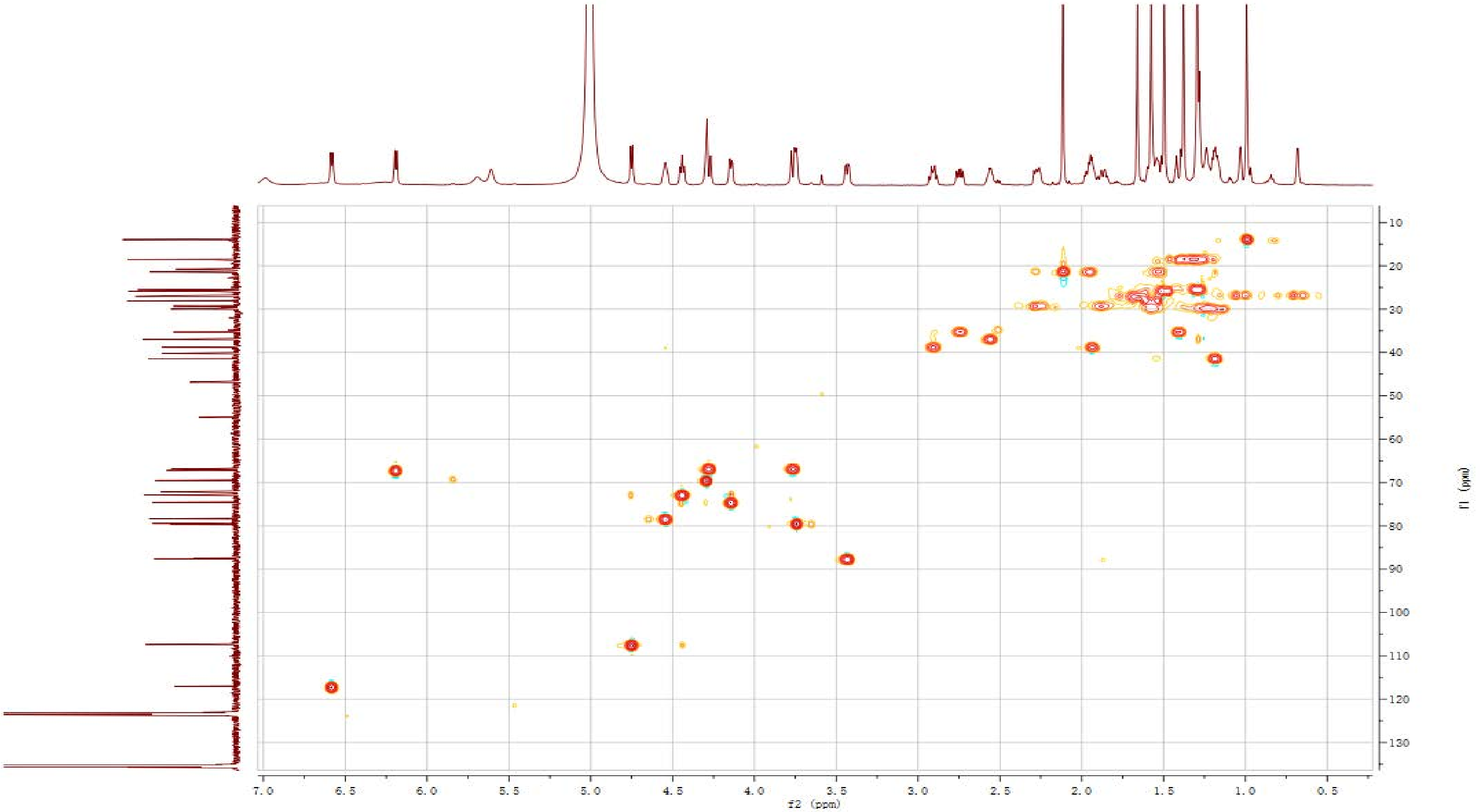

Figure S4. HMBC (600 Hz) Spectrum of cimyunnin E (**1**) in Pyridine-d_5

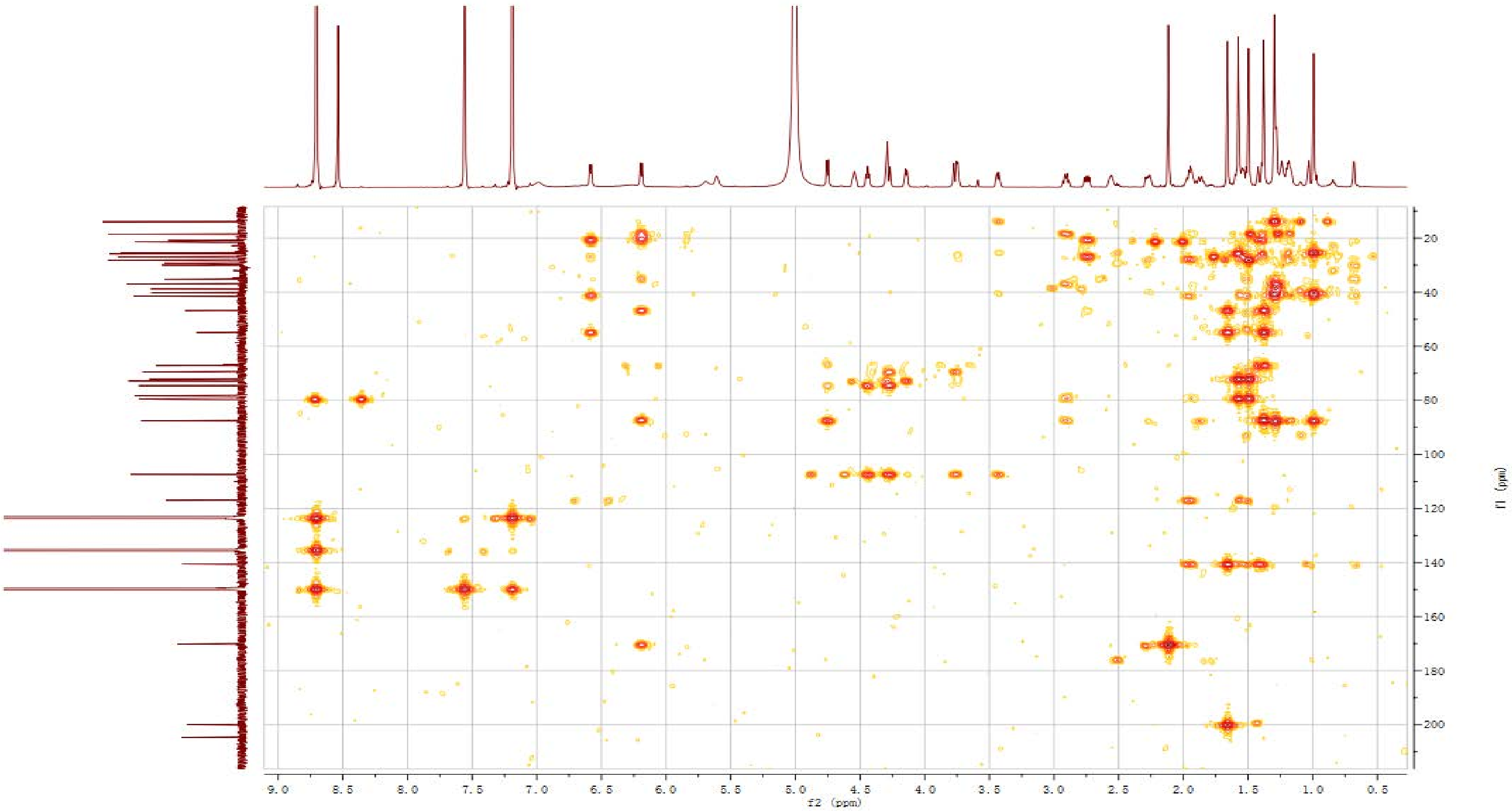

Figure S5. ^{1}H-^{1}H COSY (600 Hz) Spectrum of cimyunnin E (**1**) in Pyridine-d_5

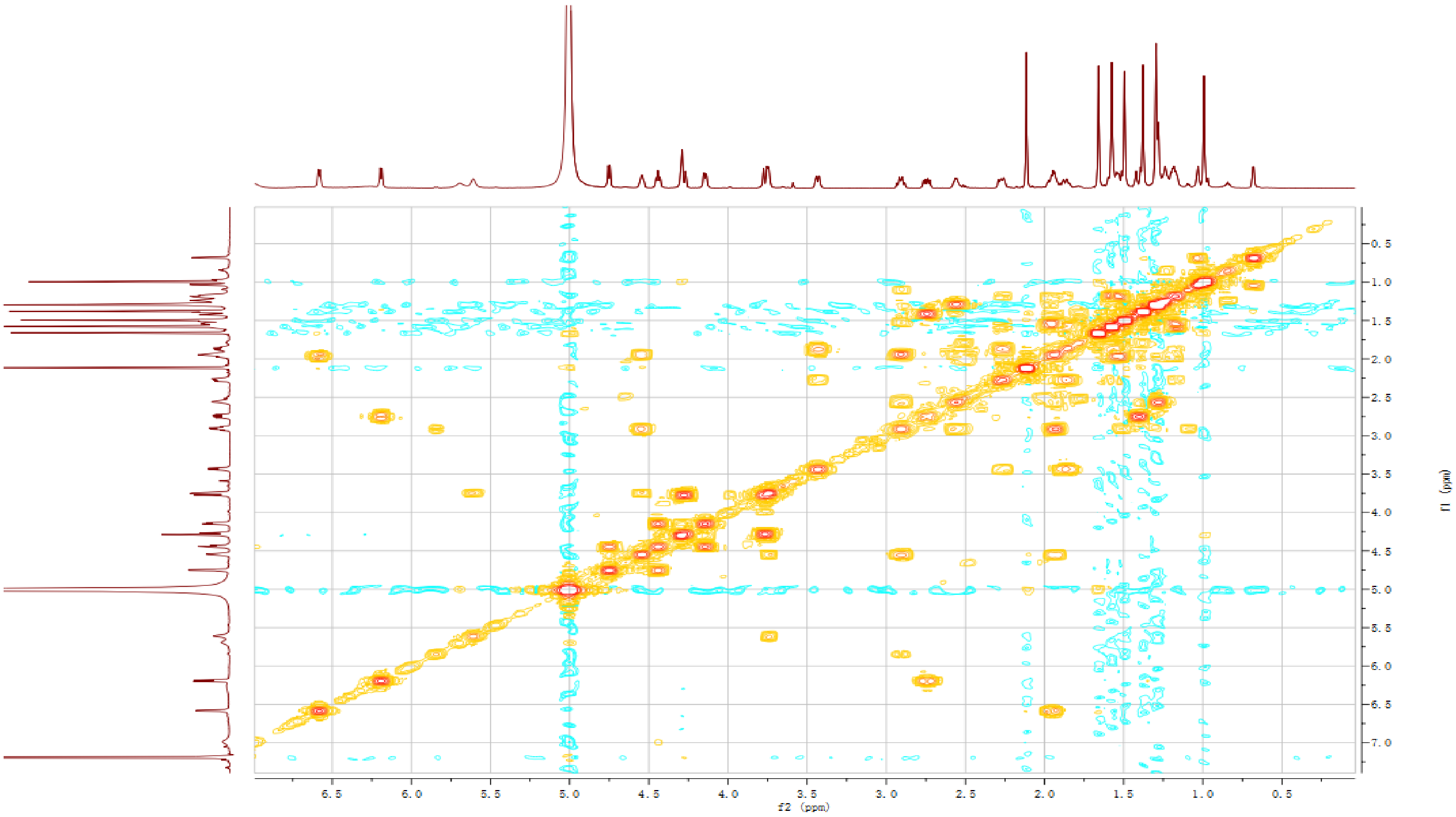

Figure S6. ROESY (600 Hz) Spectrum of cimyunnin E (**1**) in Pyridine-d_5

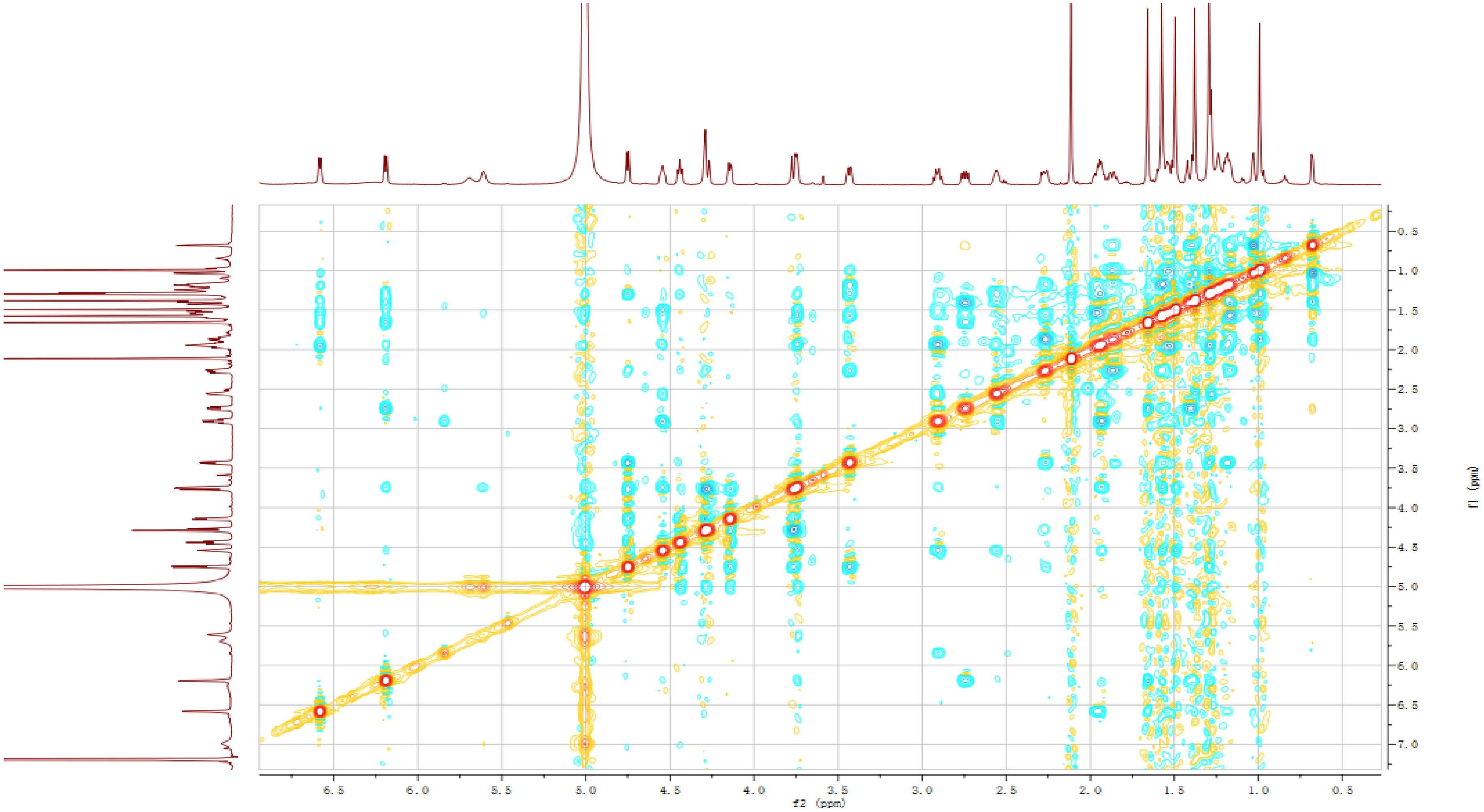

Figure S7. HREIMS of cimyunnin E (**1**)

Qualitative Analysis Report

**Data Filename**	151109ESIA9.d	**Sample Name**	kys51
**Sample Type**	Sample	**Position**	
**Instrument Name**	Agilent G6230 TOF MS	**User Name**	KIB
**Acq Method**	ESI.m	**Acquired Time**	11/6/2015 3:15:41 PM
**IRM Calibration Status**	Success	**DA Method**	ESI.m
**Comment**			

Sample Group **Info.**
Acquisition SW 6200 series TOF/6500 series
Version Q-TOF B.05.01 (B5125.2)

User Spectra

Fragmentor Voltage	Collision Energy	Ionization Mode
200	0	ESI

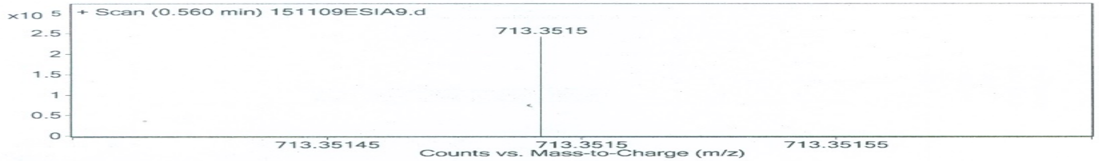

Peak List

m/z	z	Abund	Formula	Ion
121.0509		17994.03		
713.3515	1	242235.91	C37 H54 Na O12	M+
714.3541	1	89284.27	C37 H54 Na O12	M+
715.3575	1	20776.43	C37 H54 Na O12	M+
745.3762	1	33944.5		
922.0098	1	30175.05		
1403.7114	1	60208.43		
1404.7151	1	47280.52		
1405.7178	1	19297.12		
1435.7365	1	19889.5		

Formula Calculator Element Limits

Element	Min	Max
C	0	200
H	0	400
O	5	15
Na	1	1

Formula Calculator Results

Formula	CalculatedMass	CalculatedMz	Mz	Diff. (mDa)	Diff. (ppm)	DBE
C37 H54 Na O12	713.3513	713.3507	713.3515	-0.5	-0.8	10.5000

--- End Of Report ---

Agilent Technologies Page 1 of 1 Printed at: 3:32 PM on: 11/6/2015

Figure S8. UV spectrum of cimyunnin E (**1**)

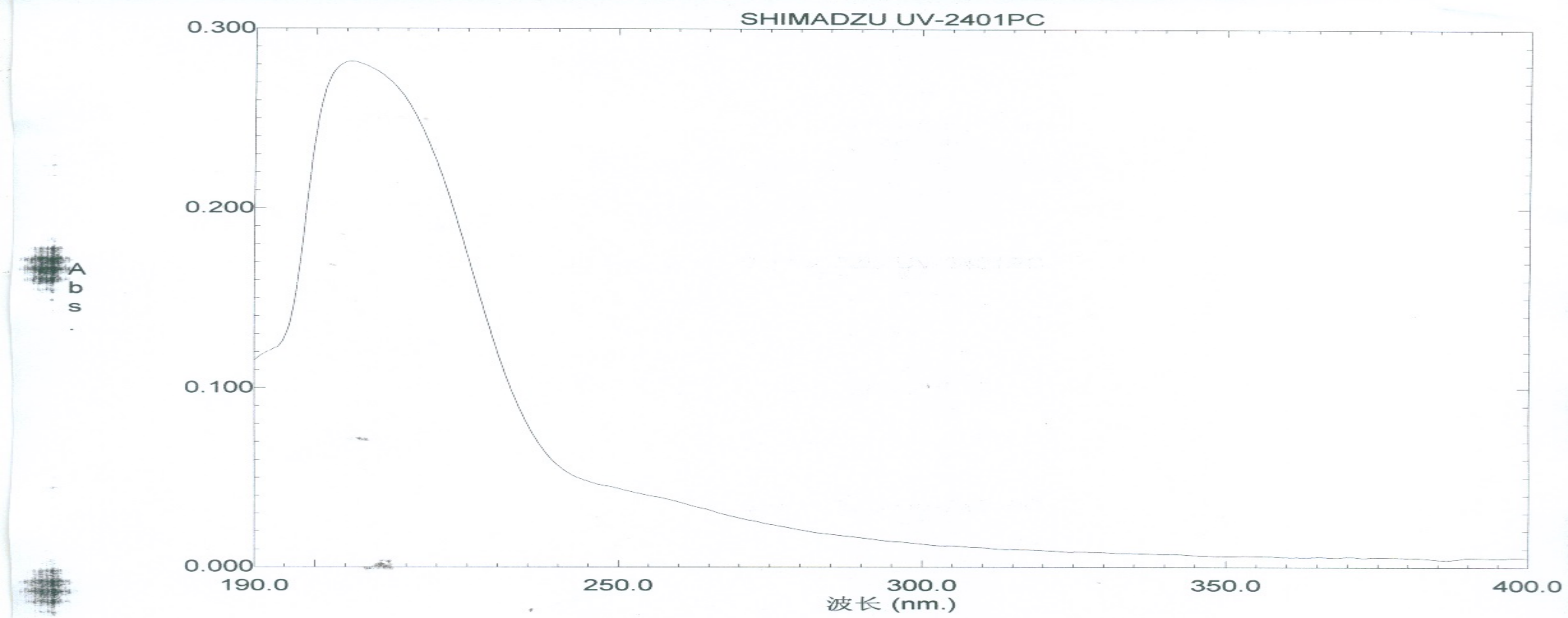

文件名: KYS51

KYS51

创建于: 20:51 15-11-03
数据: 原始

样品浓度：0.0300毫克/毫升
溶剂：甲醇

测量模式: Abs.
扫描速度: 中速
狭缝: 5.0
采样间隔: 0.2

否.	波长 (nm.)	Abs.
1	205.60	0.2816
2	250.00	0.0439

Figure S9. IR spectrum of cimyunnin E (**1**)

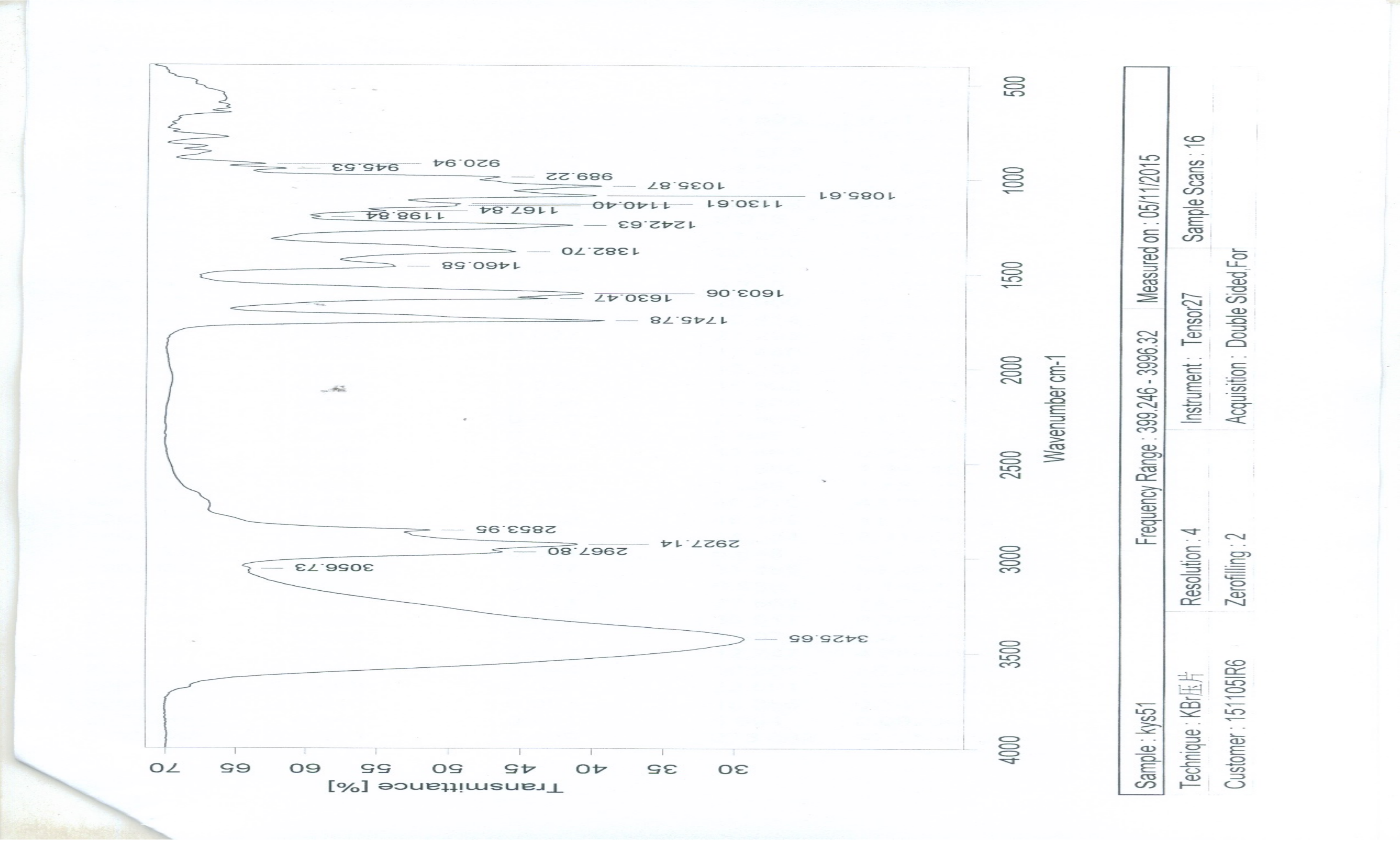

Figure S10. CD spectrum of cimyunnin E (**1**)

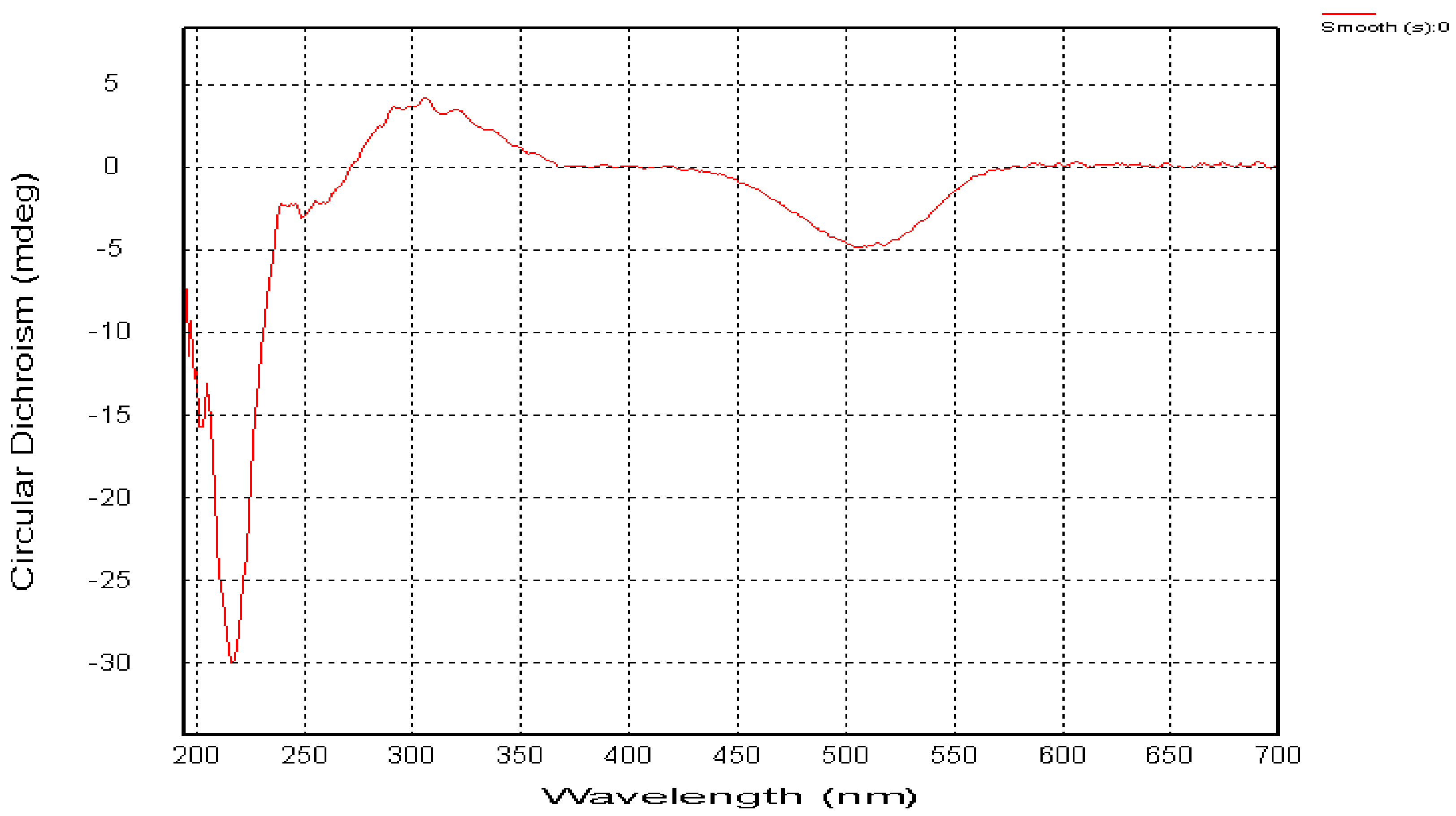

Figure S11. $[\alpha]_D$ value of cimyunnin E (**1**)

Optical rotation measurement

Model : P-1020 (A060460638)

No.	Sample	Mode	Data	Monitor / Blank	Temp. / Cell / Temp Point	Date / Comment / Sample Name	Light / Filter / Operator	Cycle Time / Integ Time
No.1	16 (1/3)	Sp.Rot	-304.0000	-0.0304	23.6	Thu Oct 29 19:24:26 2015	Na	2 sec
				0.0000	10.00	0.00100g/mL MeOH	589nm	10 sec
					Cell	KYS51		
No.2	16 (2/3)	Sp.Rot	-302.0000	-0.0302	23.6	Thu Oct 29 19:24:39 2015	Na	2 sec
				0.0000	10.00	0.00100g/mL MeOH	589nm	10 sec
					Cell	KYS51		
No.3	16 (3/3)	Sp.Rot	-306.0000	-0.0306	23.6	Thu Oct 29 19:24:53 2015	Na	2 sec
				0.0000	10.00	0.00100g/mL MeOH	589nm	10 sec
					Cell	KYS51		

-304.0000°

Figure S12. ^{1}H (600 Hz) NMR Spectrum of cimicifine B (**2**) in Pyridine-d_5

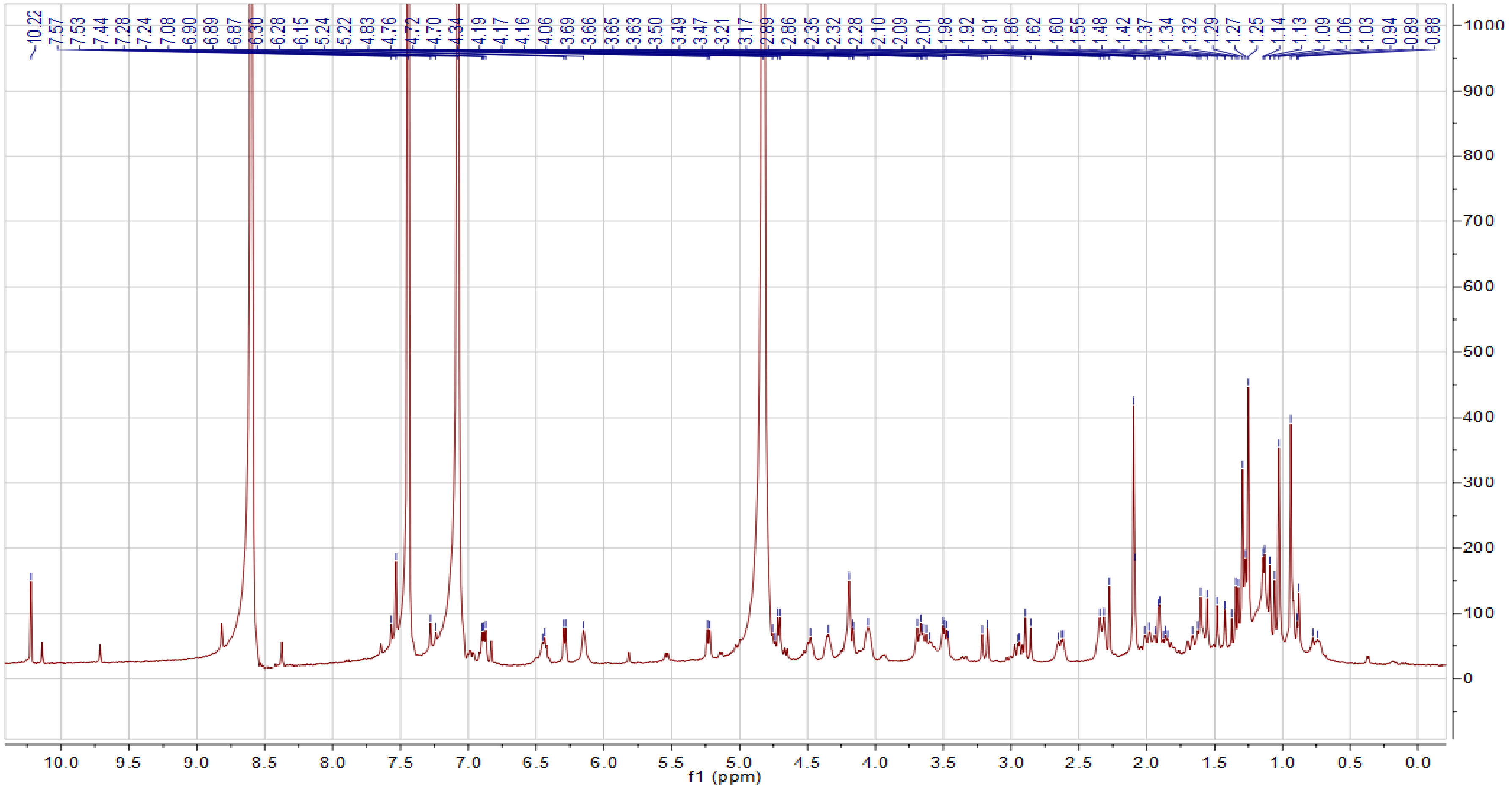

Figure S13. ^{13}C (150 Hz) NMR Spectrum of cimicifine B (**2**) in Pyridine-d_5

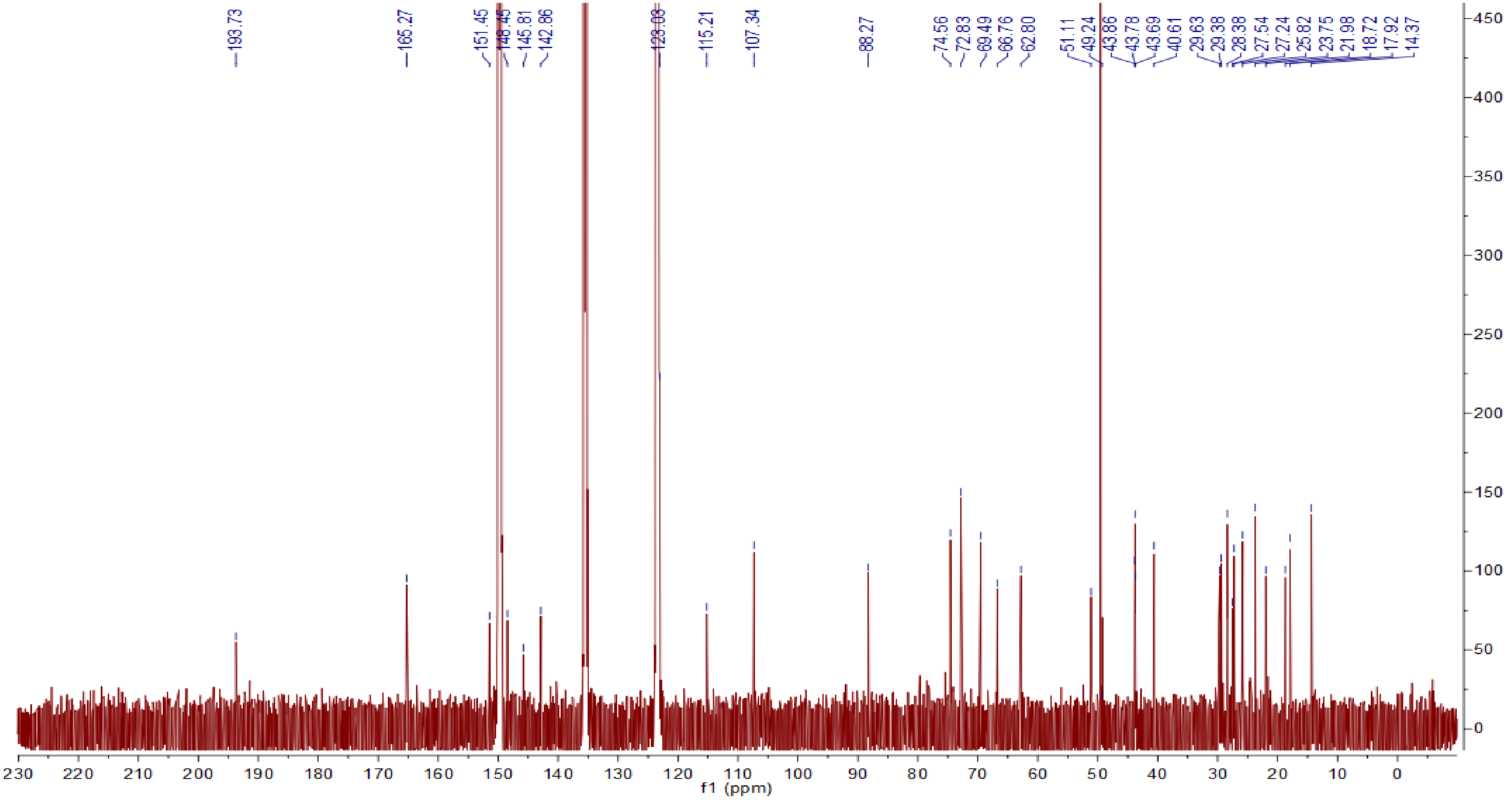

Figure S14. HSQC (600 Hz) Spectrum of cimicifine B (**2**) in Pyridine-d_5

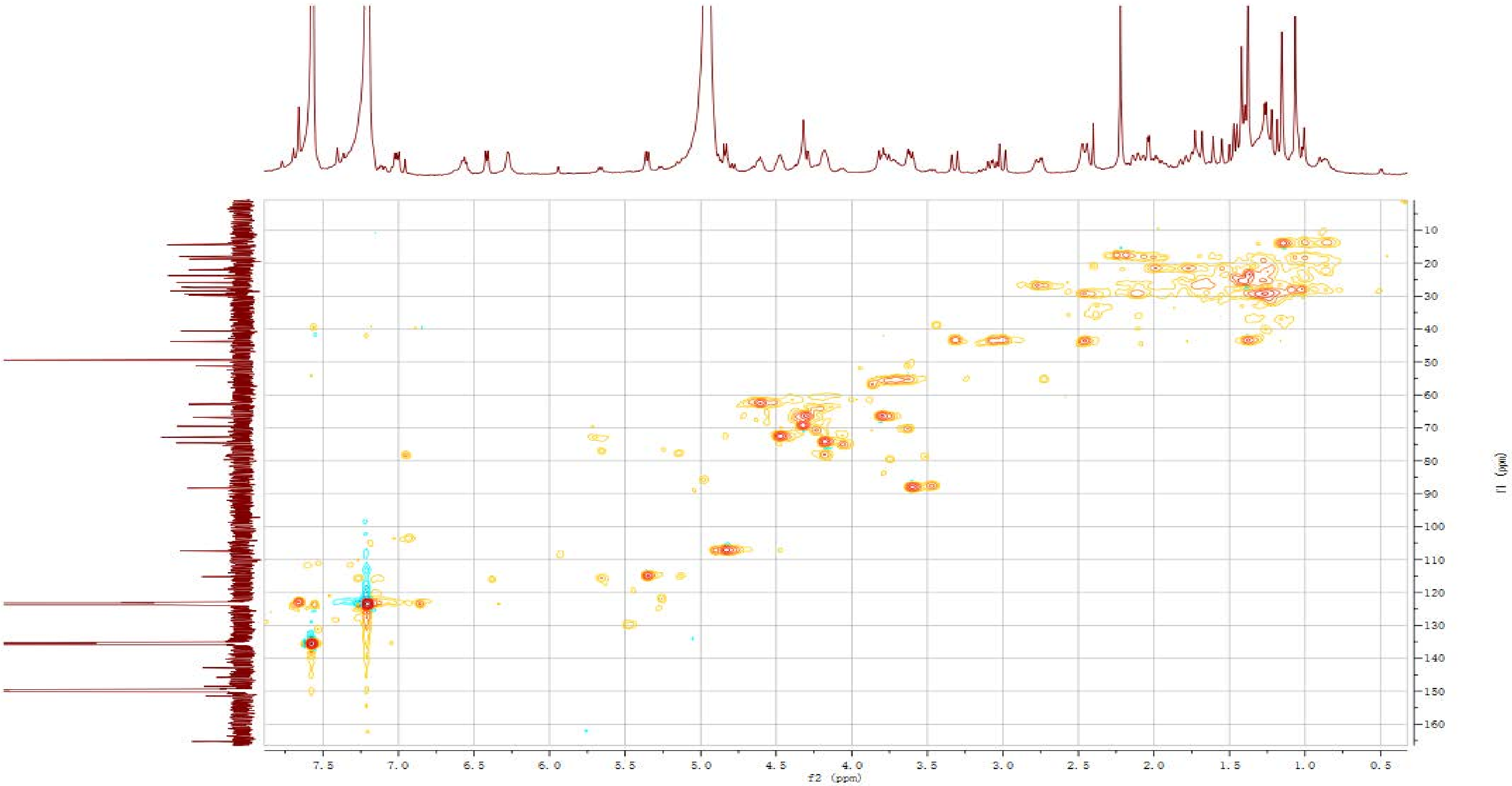

Figure S15. HMBC (600 Hz) Spectrum of cimicifine B (**2**) in Pyridine-d_5

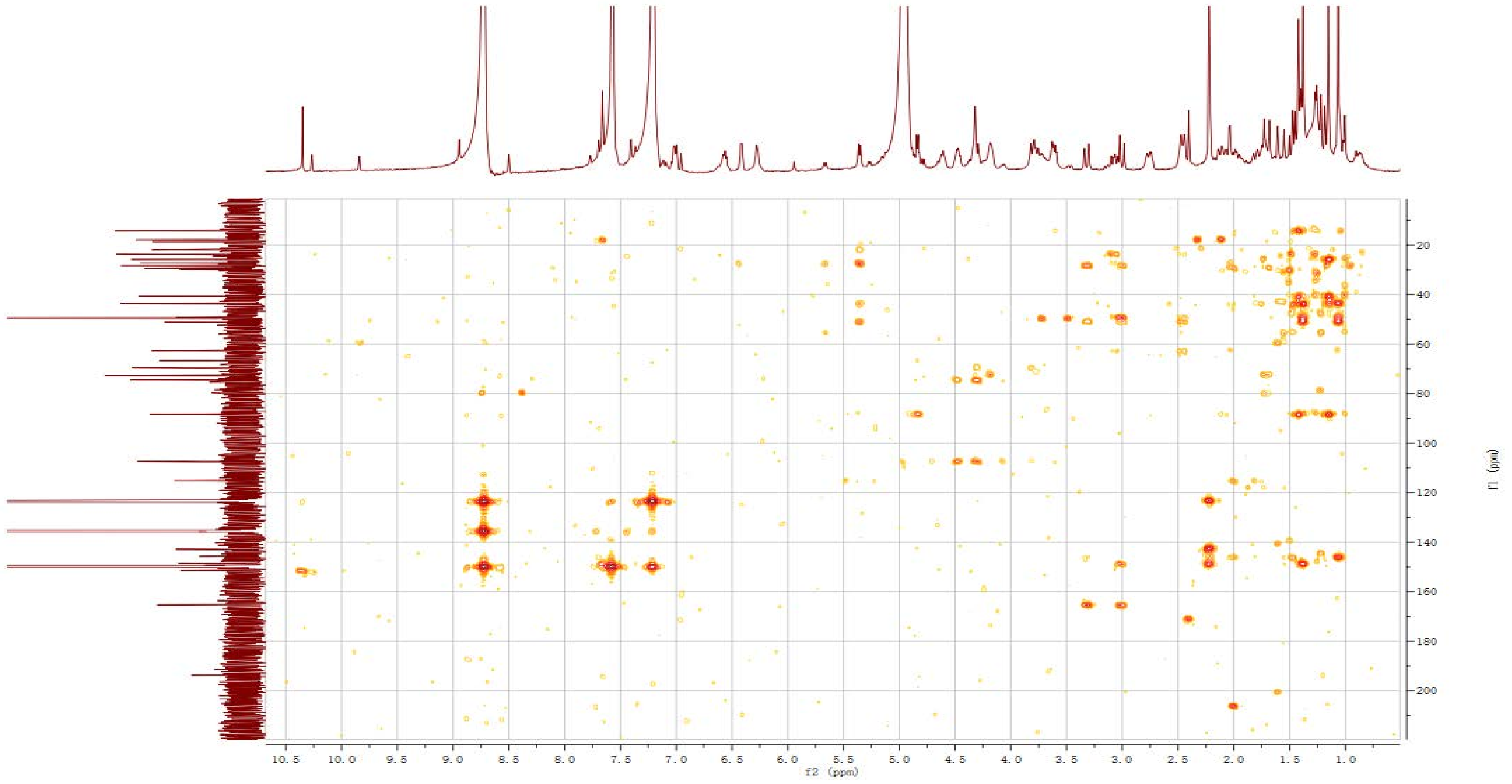

Figure S16. ^{1}H-^{1}H COSY (600 Hz) Spectrum of cimicifine B (**2**) in Pyridine-d_5

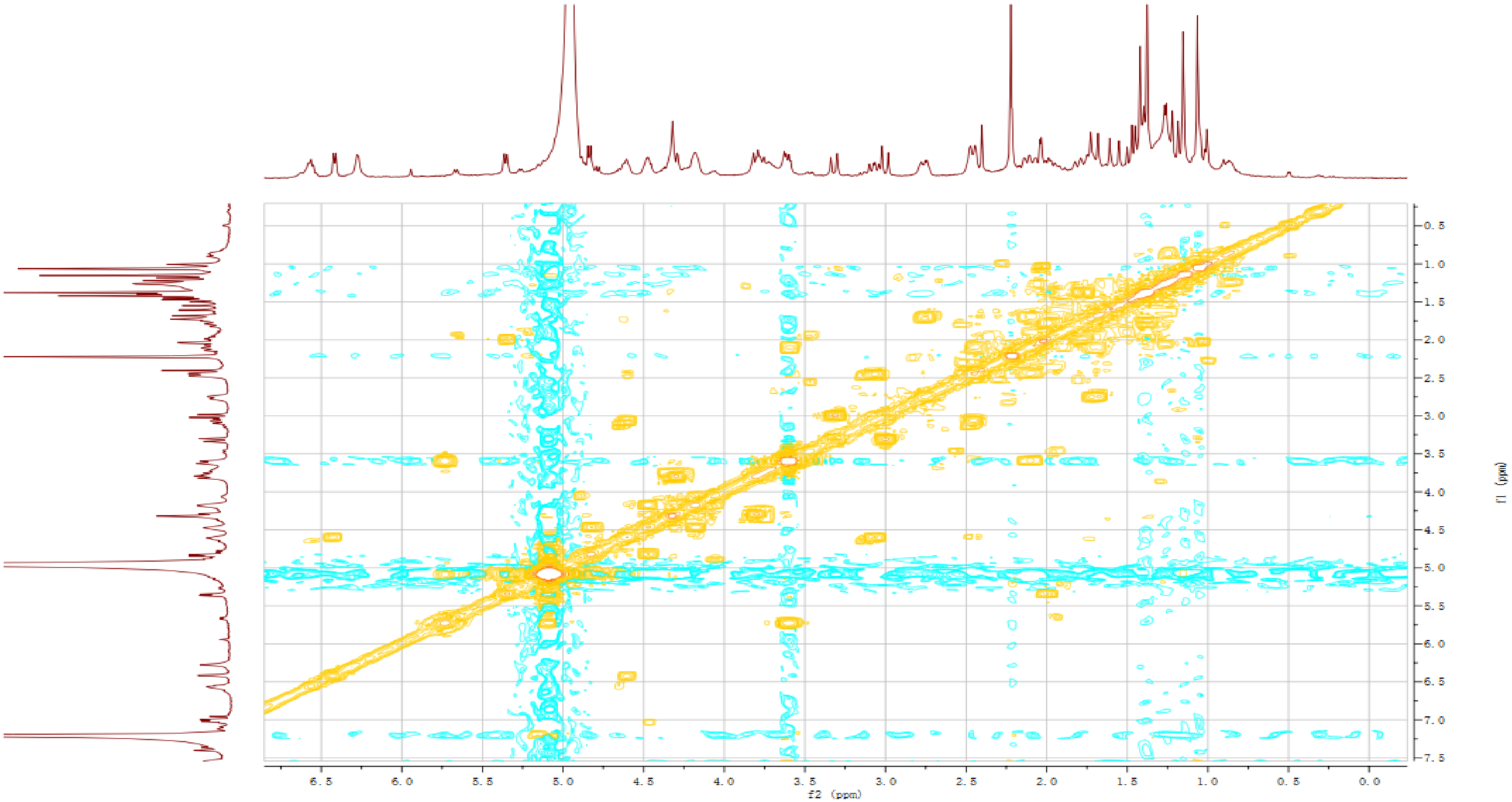

Figure S17. ROESY (600 Hz) Spectrum of cimicifine B (**2**) in Pyridine-d_5

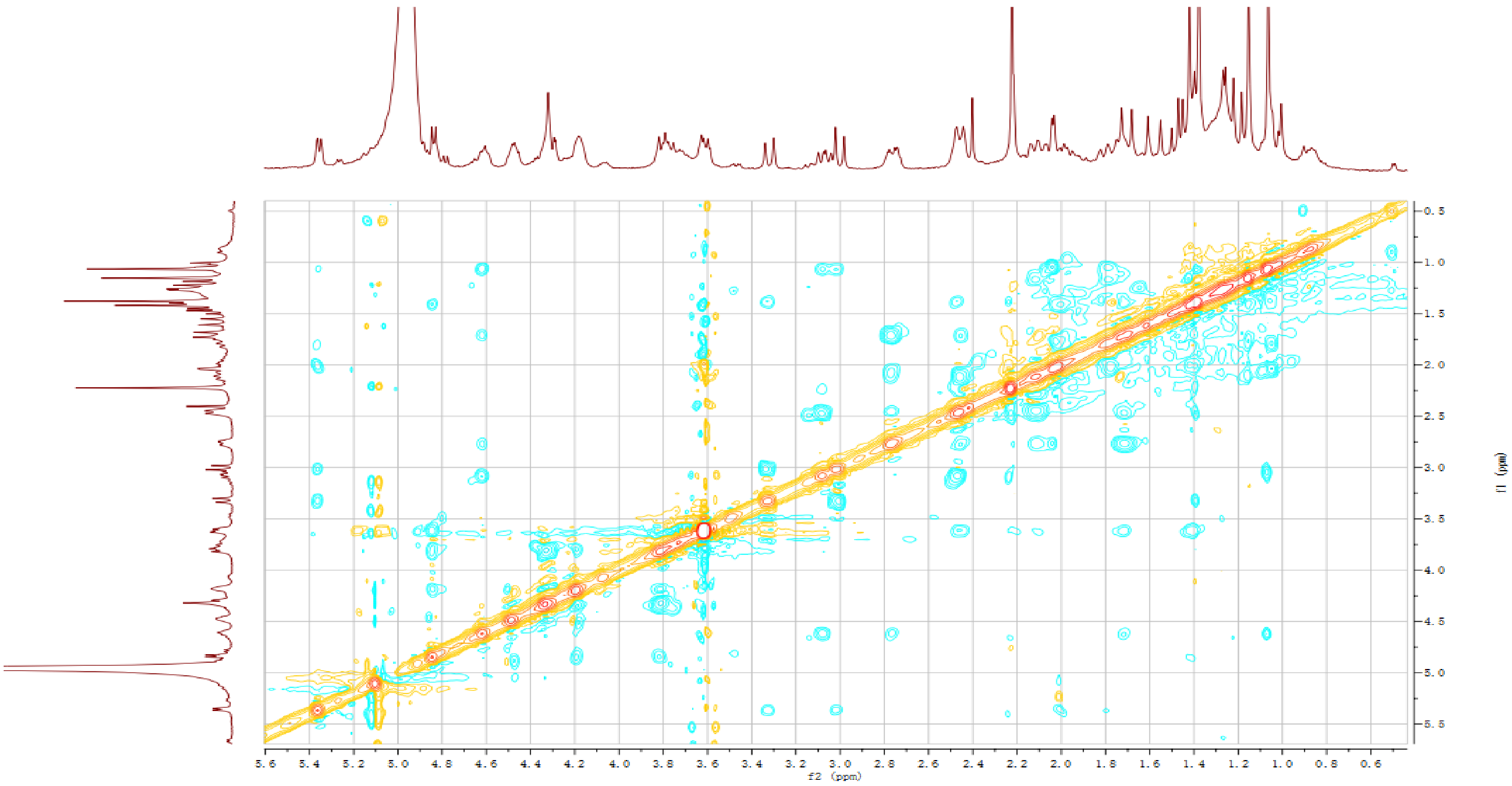

Figure S18. HREIMS of cimicifine B (**2**)

Qualitative Analysis Report

**Data Filename**	151229ESIA1.d	**Sample Name**	kys52
**Sample Type**	Sample	**Position**	
**Instrument Name**	Agilent G6230 TOF MS	**User Name**	KIB
**Acq Method**	ESI.m	**Acquired Time**	12/29/2015 3:44:31 PM
**IRM Calibration Status**	Success	**DA Method**	ESI.m
**Comment**			

Sample Group **Info.**
Acquisition SW 6200 series TOF/6500 series
Version Q-TOF B.05.01 (B5125.2)

User Spectra

Fragmentor Voltage	Collision Energy	Ionization Mode
200	0	ESI

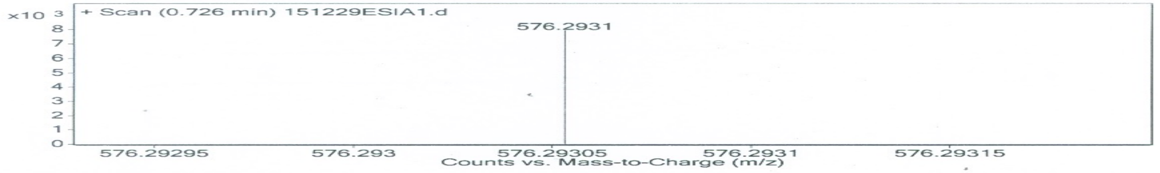

Peak List

m/z	z	Abund
299.1101	1	31643.14
301.1406		15772.27
443.3339	1	17733.49
457.3499	1	16567.32
487.3603	1	20594.22
501.3764	1	18990.18
531.3863	1	19861.49
545.4022	1	17209.74
575.4119	1	17766.5
589.4284	1	16384.11

Formula Calculator Element Limits

Element	Min	Max
C	0	200
H	0	400
O	0	13
N	1	1
Na	1	1

Formula Calculator Results

Formula	CalculatedMass	Mz	Diff.(mDa)	Diff. (ppm)	DBE
C32 H43 N Na O7	576.2937	576.2931	0.6	1.1	11.5

--- End Of Report ---

Figure S19. UV spectrum of cimicifine B (**2**)

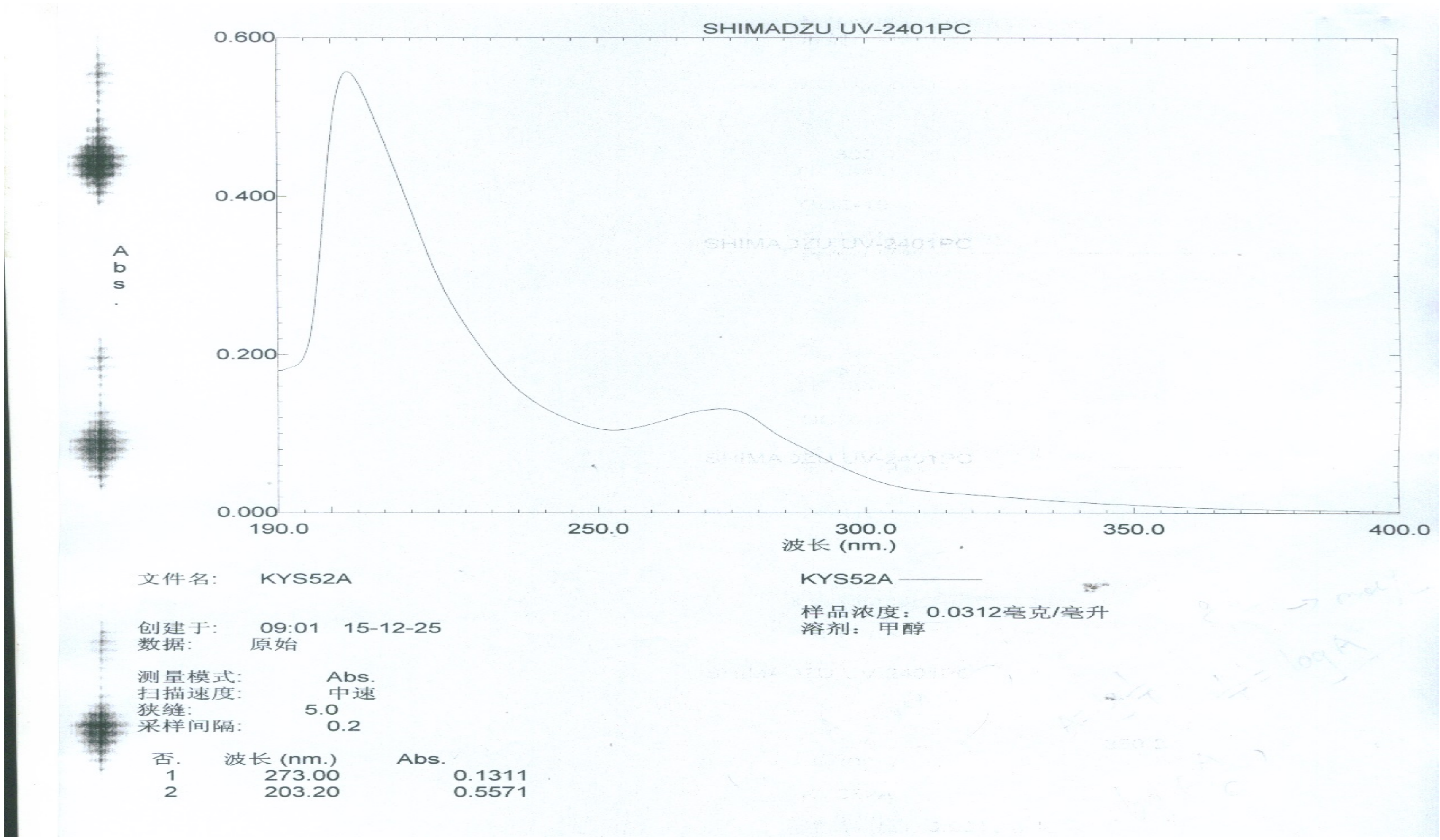

Figure S19. IR spectrum of cimicifine B (**2**)

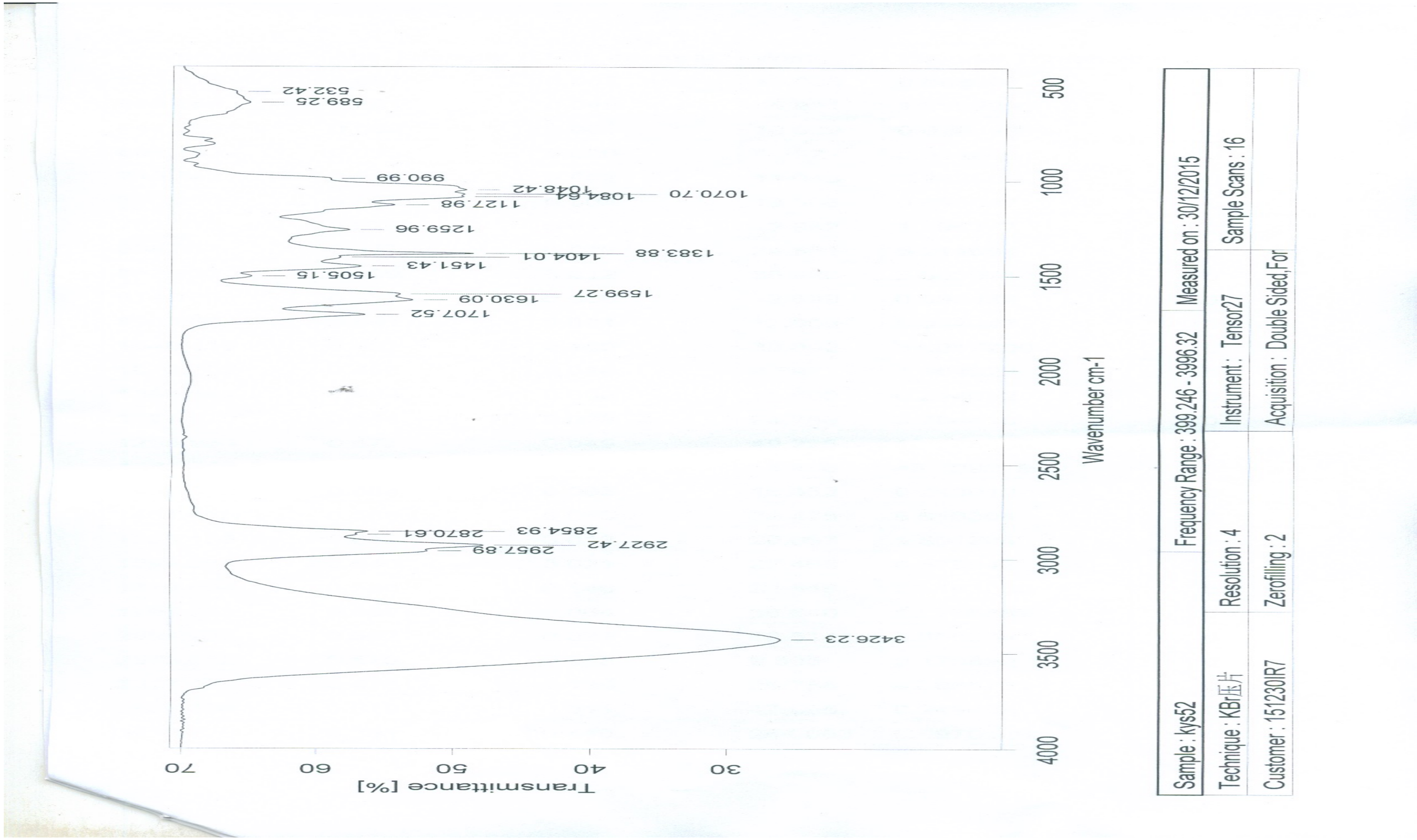

Figure S20. $[\alpha]_{D}$ value of cimicifine B (**2**)

Optical rotation measurement

Model : P-1020 (A060460638)

No.	Sample	Mode	Data	Monitor<br>Blank	Temp.<br>Cell<br>Temp Point	Date<br>Comment<br>Sample Name	Light<br>Filter<br>Operator	Cycle Time<br>Integ Time
No.1	3 (1/3)	Sp.Rot	-4.7690	-0.0031<br>0.0000	19.0<br>50.00<br>Cell	Tue Dec 22 16:18:30 2015<br>0.00130g/mL MeOH<br>KYS52A	Na<br>589nm	2 sec<br>10 sec
No.2	3 (2/3)	Sp.Rot	-4.9230	-0.0032<br>0.0000	19.0<br>50.00<br>Cell	Tue Dec 22 16:18:43 2015<br>0.00130g/mL MeOH<br>KYS52A	Na<br>589nm	2 sec<br>10 sec
No.3	3 (3/3)	Sp.Rot	-4.9230	-0.0032<br>0.0000	19.0<br>50.00<br>Cell	Tue Dec 22 16:18:57 2015<br>0.00130g/mL MeOH<br>KYS52A	Na<br>589nm	2 sec<br>10 sec

-4.8718°

Figure S21. Structure of 17*R*, 20*S*, 23*S*, and 24*R* counterpart of **1** and unlikely happened ROESY (◄- - - -►) correlations

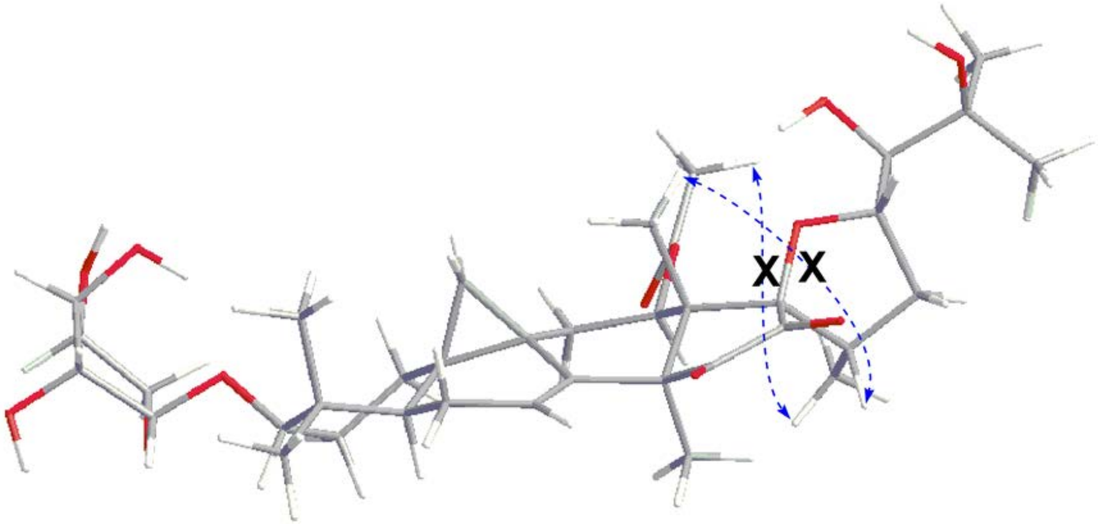

Table S1. Dose-related inhibitions of compounds **1** and **2** on acetylcholinesterase (AChE)

Compounds	Concentration (μM)	Inhibitory ratio (%)	IC_{50} (μM)
	100	85.844 ± 3.004	
	50	84.211 ± 2.912	
	30	83.290 ± 4.346	
**1**	10	74.776 ± 3.849	1.58
	3	57.700 ± 2.735	
	1	27.155 ± 2.559	
	0.2	1.578 ± 0.564	
	100	84.443 ± 4.879	
	50	84.312 ± 3.230	
	30	82.731 ± 4.276	
**2**	10	71.494 ± 3.458	3.87
	3	32.850 ± 4.021	
	1	3.851 ± 1.032	
	0.2	0.983 ± 0.257	
	2	83.776 ± 0.287	
	1	77.690 ± 2.950	
	0.5	65.138 ± 4.989	
**Tacrine**	0.2	49.498 ± 1.985	0.16
	0.04	13.553 ± 1.239	
	0.008	2.884 ± 4.541	
	0.0016	-4.744 ± 3.439	

All the data were represented as mean ± SEM (n=3). 2% DMSO as the negative control (NC group). The percentage inhibition was calculated as follows: % inhibition = (NC - S)/NC × 100 (NC is the activity of the enzyme without test compound and with 2% DMSO and S is the activity of enzyme with test compound and the final concentration of DMSO is 0.1%).

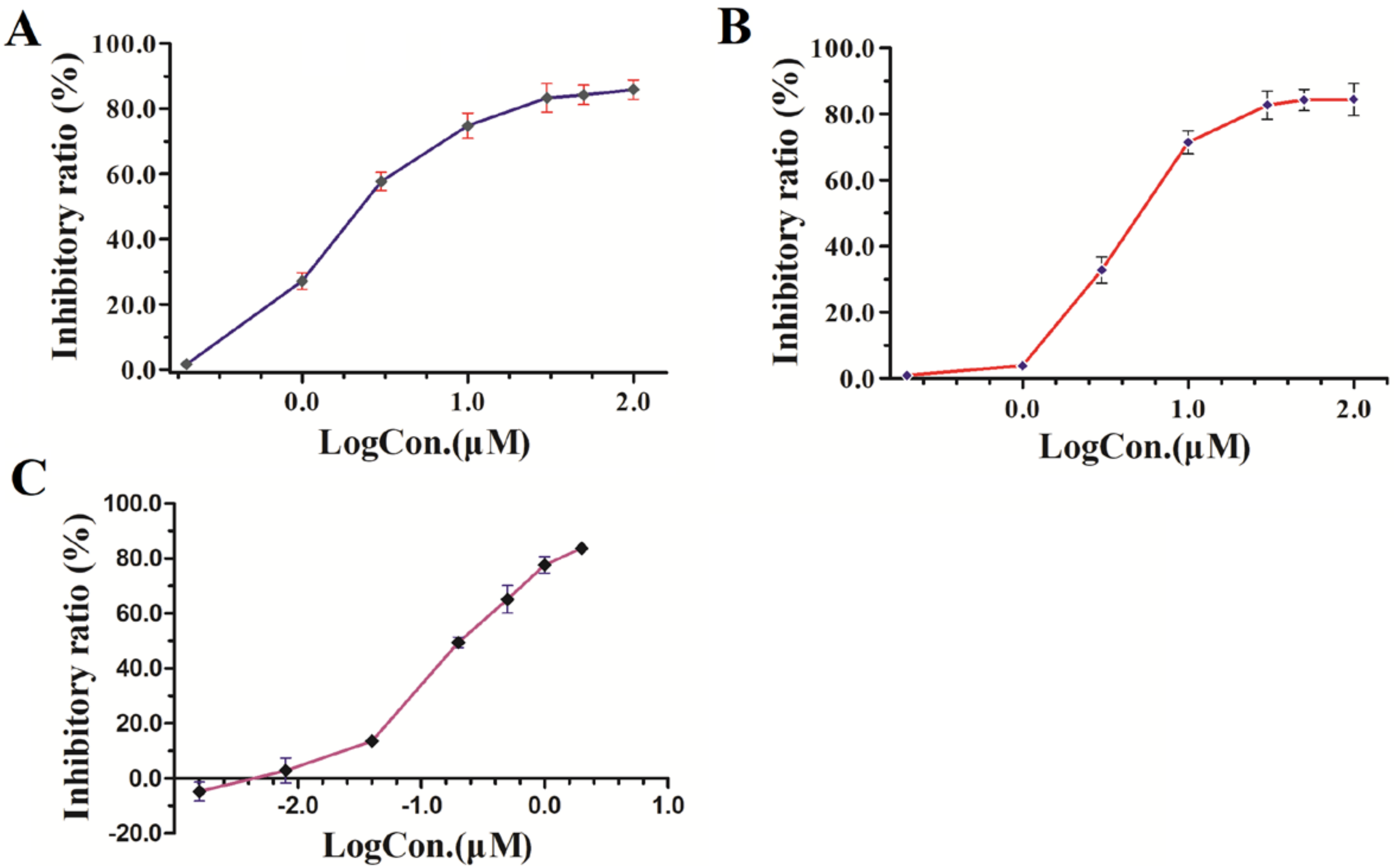

Figure S22. Dose-response relationships of compounds **1** (A), **2** (B), and Tacrine (C). Data points represent mean ± SEM of three measurements. The solid curves represent a fit to the Hill equation with IC_{50} value of 1.58 (A), 3.87 (B) and 0.17 (C) μM, respectively, and a Hill coefficient of 1.2 (A), 1.9 (B), and 0.9 (C), respectively.

Table S2. The effects of compounds **1** and **2** on the NGF mediated neurite outgrowth of PC12 cells

Groups	Blank	Negative Control (5ng/mL NGF)	Positive Control (50ng/mL NGF)	1 (10μM)	2 (10μM)
Differentiation rates (%)	0.34%	4.17%	19.18%	15.34%	11.72%

Figure S23. Represented pictures of the NGF mediated neurite outgrowth of PC12 cells. **1.** Blank; **2.** Negative Control (5ng/mL NGF); **3.** Positive Control (50ng/mL NGF); **4.** Compound **1** (10μM); **5.** Compound **2** (10μM). All pictures were took after 72 h incubation.

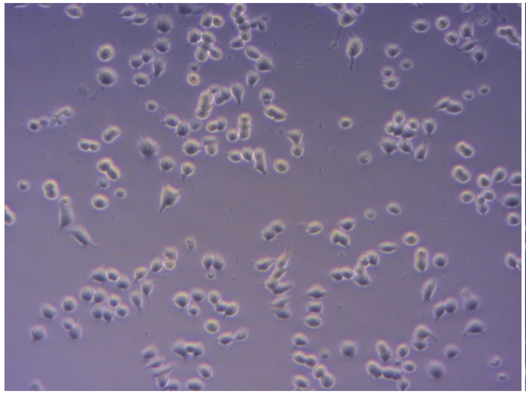

1

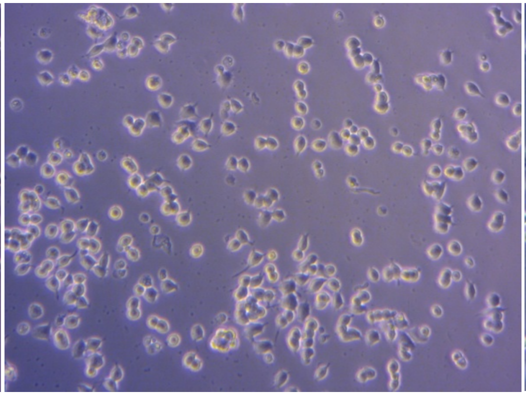

2

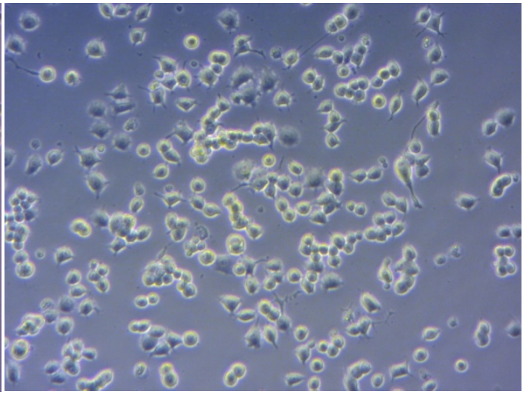

3

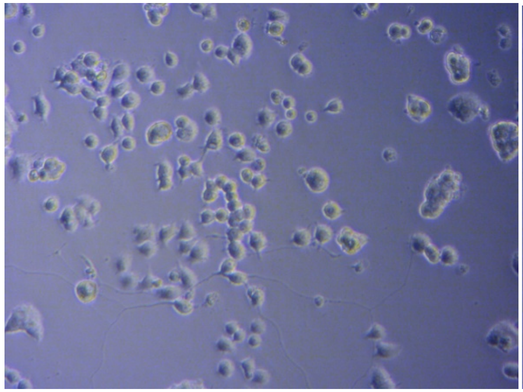

4

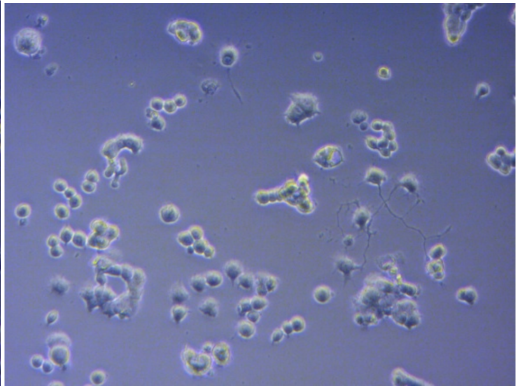

5

Computational methods of 1

1.1 Conformational analysis

Conformational analysis was initially performed using Confab[1] with systematic search at MMFF94 force field for the two configurations **a** and **b** of compound **1** (**Figure S23**). The energies and populations of all dominative conformers were provided in **Table S3**.

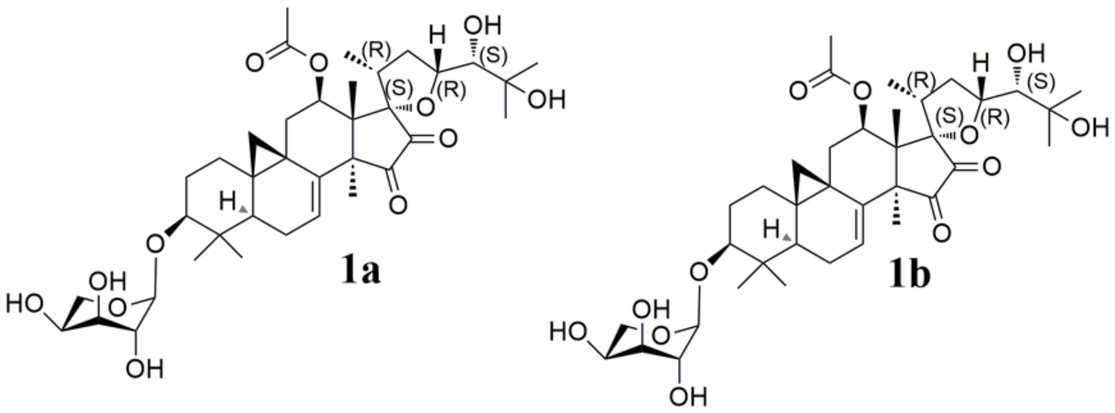

Figure S24. Chemical structure of configurations **a** and **b** of compound **1**

1.2 ECD calculation

The theoretical calculationswere carried out using Gaussian 09[2]. Structures were first optimized at PM6 using semi-empirical theory method and then optimized at HF/6-31G(d) theory level.Room-temperature equilibrium populations were calculated according to Boltzmann distribution

law (eq. **1**). The conformers with Boltzmann-population of over 1% were chosen and further optimized at B3LYP/6-311G(d,p) in methanolusing the IEFPCM model (**Table S3**). Vibrational frequency analysis confirmed the stable structures.

$$\frac{N_i}{N} = \frac{g_i e^{-\frac{E_i}{k_B T}}}{\sum g_i e^{-\frac{E_i}{k_B T}}} \qquad \textbf{(1)}$$

where N_i is the number of conformer i with energy E_i and degeneracy g_i at temperature *T*, and k_B is Boltzmann constant.

Under the same condition, the ECD calculation was conducted using Time-dependent Density functional theory (TD-DFT). Rotatory strengths for a total of 30 excited states were calculated. The ECD spectrum was simulated in SpecDis[3] by overlapping Gaussian functions for each transition according to (eq. **2**):

$$\Delta\varepsilon(E) = \frac{1}{2.297\times 10^{-39}} \times \frac{1}{\sqrt{2\pi\sigma}} \sum_{i}^{A} \Delta E_i R_i e^{-\left(\frac{E-E_i}{2\sigma}\right)^2} \qquad \textbf{(2)}$$

where σ represents the width of the band at 1/*e* height, and ΔE_i and R_i are the excitation energies and rotatory strengths for transition *i*, respectively. Parameters of σ and UV-shift for enantiomers were 0.5eV and 1 nm, respectively.

1.3 References

1. Noel M OBoyle, Tim V, ermeersch, Christopher J Flynn, Anita R Maguire Maguire, and Geoffey R Hutchison. Confab - systematic generation of diverse low-energy conformers. *Journal of Cheminformatics*, 2011, **3**, 3-8.
2. M. J. Frisch, G. W. Trucks, H. B. Schlegel, G. E. Scuseria, M. A. Robb, J. R. Cheeseman, G. Scalmani, V. Barone, B. Mennucci, G. A. Petersson, H. Nakatsuji, M. Caricato, X. Li, H. P. Hratchian, A. F. Izmaylov, J. Bloino, G. Zheng, J. L. Sonnenberg, M. Hada, M. Ehara, K. Toyota, R. Fukuda, J. Hasegawa, M. Ishida, T. Nakajima, Y. Honda, O. Kitao, H. Nakai, T. Vreven, J. A. Montgomery, Jr., J. E. Peralta, F. Ogliaro, M. Bearpark, J. J. Heyd, E. Brothers, K. N. Kudin, V. N. Staroverov, R. Kobayashi, J. Normand, K. Raghavachari, A. Rendell, J. C. Burant, S. S. Iyengar, J. Tomasi, M. Cossi, N. Rega, J. M. Millam, M. Klene, J. E. Knox, J. B. Cross, V. Bakken, C. Adamo, J. Jaramillo, R. Gomperts, R. E. Stratmann, O. Yazyev, A. J. Austin, R. Cammi, C. Pomelli, J. W. Ochterski, R. L. Martin, K. Morokuma, V. G. Zakrzewski, G. A. Voth, P. Salvador, J. J. Dannenberg, S. Dapprich, A. D. Daniels, O. Farkas, J. B. Foresman, J. V. Ortiz, J. Cioslowski, and D. J. Fox. Gaussian 09 Revision D.01. Gaussian Inc. Wallingford CT 2009.
3. Torsten Bruhn, Anu Schaumlffl, Anu Schaumlffl, Yasmin Hemberger, Yasmin Hemberger, Gerhard Bringmann, and Gerhard Bringmann. Specdis: quantifying the comparison of calculated and experimental electronic circular dichroism spectra. *Chirality*, 2013, **25**, 243-249.

Energies and Coordinates

2.1 Energies at MMFF94 force field

Systematic conformational search was performed by Confab program at MMFF94 force field. Conformers for each configuration were obtained with filtration by RMSD threshold of 2.0 Å.

Table S3. Energies of compound **1** at MMFF94 force field.

Configuration	Conformer	Energy (kcal/mol)
a	1	323.27
	2	326.43
	3	329.59
	4	334.05
	5	343.68
	6	344.90
	7	346.19
	8	347.20
	9	348.80
	10	351.99
	11	358.27
	12	363.11
b	1	339.44

2	345.35
3	348.36
4	354.33
5	359.80
6	363.16
7	369.10
8	371.19

2.2 Energies at B3LYP theory level

Structures for ECD calculations were optimized at B3LYP/6-311G(d,p) in methanol and conformers with Boltzmann-based population high than 1% were retained.

Table S4. Energies of compound **1** at B3LYP/6-311G(d,p) in methanol.

Configuration	Conformer	Structure	E (Hartree)	E (kcal/mol)	Population (%)
a	2	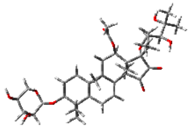	-2345.70091427	-1471949.54	74.04
a	9	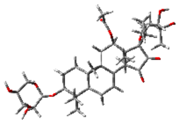	-2345.69992561	-1471948.91	25.96
b	1	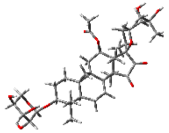	-2345.70079978	-1471949.46	72.17

b	4	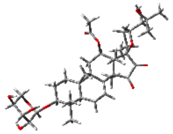	-2345.69990069	-1471948.90	27.83

2.3 Coordinates at B3LYP theory level

Table S5. Standard orientations of compound **1** at B3LYP/6-311G(d,p) level in methanol.

Conformer a-2

Center	Atomic	Atomic	Coordinates (Angstroms)		
Number	Number	Type	X	Y	Z
1	8	0	-4.496612	-0.037145	0.651423
2	8	0	-2.226570	2.157494	-1.257128
3	8	0	6.040172	-0.431911	0.231225
4	8	0	6.853357	1.192823	1.728855
5	8	0	-2.193590	3.681789	0.408191
6	8	0	8.926738	-0.828627	2.276618
7	8	0	8.655412	0.183966	-1.214638
8	8	0	9.585390	2.557965	-0.347567
9	6	0	0.267743	-0.217309	-0.484518
10	6	0	1.796432	-0.251803	-0.335128
11	6	0	1.185367	0.001787	-1.687561
12	6	0	2.441743	-1.607609	-0.018870
13	6	0	-1.944244	-1.438096	-0.085533
14	6	0	-0.451318	-1.536949	-0.380079
15	6	0	-2.582506	-0.212174	-0.881833
16	6	0	-0.493629	0.989783	0.065505
17	6	0	2.483072	0.891974	0.400560
18	6	0	-2.000802	1.080368	-0.297334

19	6	0	-4.142282	-0.382724	-0.705624
20	6	0	3.987302	-1.631679	-0.297775
21	6	0	1.690854	-2.788750	-0.663106
22	6	0	-2.864472	-2.566156	-0.515388
23	6	0	-4.268844	-1.924253	-0.738273
24	6	0	0.200832	-2.699370	-0.477868
25	6	0	3.995498	0.902001	0.151776
26	6	0	4.620528	-0.449834	0.488730
27	6	0	-2.111769	-1.420944	1.476228
28	6	0	-5.232954	0.310414	-1.600245
29	6	0	-2.229993	-0.418341	-2.375702
30	6	0	-5.748979	0.696076	0.738991
31	1	0	-5.585374	1.437842	1.520420
32	6	0	4.594131	-2.942379	0.242047
33	6	0	4.323450	-1.511834	-1.798298
34	6	0	-5.897785	1.317174	-0.642907
35	6	0	-4.906738	0.949468	-2.951382
36	6	0	-2.298114	3.414956	-0.766948
37	6	0	6.867631	-0.171569	1.330868
38	6	0	8.287923	-0.670023	1.007846
39	6	0	-2.535308	4.417328	-1.865544
40	6	0	9.100074	0.297477	0.132686
41	6	0	7.470684	2.082345	0.789692
42	6	0	8.950210	1.735573	0.633541
43	1	0	1.223661	-0.778300	-2.438352

44	1	0	1.238429	1.008537	-2.089468
45	1	0	2.333439	-1.734587	1.069166
46	1	0	-0.410964	1.005685	1.156194
47	1	0	-0.023100	1.909291	-0.277345
48	1	0	2.067525	1.857775	0.108945
49	1	0	2.298379	0.783129	1.477502
50	1	0	-2.561058	1.338237	0.597852
51	1	0	1.928940	-2.857382	-1.731437
52	1	0	2.041280	-3.726023	-0.225889
53	1	0	-0.352268	-3.628515	-0.423609
54	1	0	4.205384	1.148569	-0.893589
55	1	0	4.469136	1.671057	0.765215
56	1	0	4.473606	-0.647569	1.560205
57	1	0	-3.151145	-1.379808	1.793220
58	1	0	-1.596138	-0.567036	1.913712
59	1	0	-1.649542	-2.330114	1.863644
60	1	0	-5.969802	-0.472170	-1.792244
61	1	0	-2.413075	0.481913	-2.951044
62	1	0	-2.827160	-1.223071	-2.818420
63	1	0	-1.187251	-0.693229	-2.505761
64	1	0	5.403925	-1.421933	-1.924838
65	1	0	3.855817	-0.643424	-2.264536
66	1	0	5.682721	-2.908950	0.170957
67	1	0	4.252090	-3.808938	-0.327366
68	1	0	4.326063	-3.099424	1.292088

69	1	0	3.996590	-2.399888	-2.344031
70	1	0	-5.374438	2.276739	-0.676390
71	1	0	-6.936257	1.483726	-0.924784
72	1	0	-5.826003	1.396272	-3.340953
73	1	0	-4.565659	0.223365	-3.689417
74	1	0	-4.162594	1.740384	-2.861205
75	1	0	6.513528	-0.707697	2.216936
76	1	0	8.200589	-1.628399	0.486345
77	1	0	-2.357126	5.421306	-1.485789
78	1	0	-3.571245	4.343428	-2.206777
79	1	0	-1.888834	4.212952	-2.719744
80	1	0	10.159906	0.010925	0.198331
81	1	0	6.972957	2.029980	-0.182814
82	1	0	7.344414	3.086230	1.198776
83	1	0	9.446972	1.822504	1.605755
84	1	0	9.781882	-1.247123	2.129278
85	1	0	9.001783	0.960888	-1.673262
86	1	0	9.510073	3.480681	-0.081246
87	8	0	-5.283157	-2.574244	-0.820723
88	8	0	-2.673167	-3.749880	-0.629059
89	8	0	-7.256232	1.081327	3.135759
90	1	0	-7.897895	1.525604	3.701135
91	8	0	-7.523589	-0.946626	0.163207
92	6	0	-8.950259	-0.577051	2.614198
93	6	0	-7.980029	0.467695	2.049734

94	6	0	-6.878373	-0.241471	1.221470
95	1	0	-6.399251	-0.948536	1.910741
96	6	0	-8.742206	1.537207	1.258981
97	1	0	-9.478542	-1.095863	1.813535
98	1	0	-9.689395	-0.090013	3.257508
99	1	0	-8.407101	-1.313659	3.211452
100	1	0	-9.200422	1.109819	0.364796
101	1	0	-8.082613	2.354215	0.961576
102	1	0	-9.538753	1.958100	1.879825
103	1	0	-6.908523	-1.610793	-0.181707

Conformer a-9

Center	Atomic	Atomic	Coordinates (Angstroms)		
Number	Number	Type	X	Y	Z
1	8	0	-4.659111	-0.067296	0.498750
2	8	0	-2.274760	2.419146	-0.971965
3	8	0	5.911382	-0.514208	0.234178
4	8	0	6.762171	1.029382	1.796813
5	8	0	-2.226802	3.672069	0.905350
6	8	0	8.751031	-1.075022	2.308227
7	8	0	8.568030	0.042046	-1.158291
8	8	0	9.558468	2.357929	-0.215473
9	6	0	0.159808	-0.087941	-0.532418
10	6	0	1.683386	-0.172027	-0.358265
11	6	0	1.109078	0.313304	-1.662346
12	6	0	2.303704	-1.571902	-0.256575

13	6	0	-2.079291	-1.320924	-0.396806
14	6	0	-0.579497	-1.393833	-0.668742
15	6	0	-2.679477	0.032890	-0.987010
16	6	0	-0.600471	1.021273	0.196576
17	6	0	2.369776	0.824671	0.566433
18	6	0	-2.095153	1.201880	-0.185855
19	6	0	-4.244992	-0.143487	-0.890105
20	6	0	3.855190	-1.577719	-0.504328
21	6	0	1.553286	-2.618898	-1.102570
22	6	0	-3.000419	-2.346188	-1.035547
23	6	0	-4.378685	-1.642753	-1.257050
24	6	0	0.060160	-2.534978	-0.941907
25	6	0	3.884634	0.854226	0.338372
26	6	0	4.488506	-0.542789	0.468023
27	6	0	-2.282262	-1.567457	1.139740
28	6	0	-5.293290	0.737981	-1.639468
29	6	0	-2.284837	0.078465	-2.484305
30	6	0	-5.708723	0.902157	0.718944
31	1	0	-5.408533	1.499738	1.582436
32	6	0	4.431932	-2.966496	-0.162797
33	6	0	4.224843	-1.232394	-1.961230
34	6	0	-5.743650	1.731150	-0.560413
35	6	0	-5.004217	1.414604	-2.979416
36	6	0	-2.311300	3.590448	-0.298557
37	6	0	6.729053	-0.321498	1.354125

38	6	0	8.136342	-0.860020	1.035814
39	6	0	-2.470334	4.754073	-1.240781
40	6	0	8.993888	0.102826	0.198827
41	6	0	7.416493	1.923021	0.887422
42	6	0	8.885403	1.531561	0.736693
43	1	0	1.156499	-0.331642	-2.531565
44	1	0	1.186884	1.371732	-1.889845
45	1	0	2.168834	-1.873700	0.793338
46	1	0	-0.549485	0.847534	1.275571
47	1	0	-0.107425	1.977051	0.030867
48	1	0	1.968734	1.830524	0.433783
49	1	0	2.168974	0.541257	1.608185
50	1	0	-2.673704	1.328615	0.726610
51	1	0	1.818112	-2.516965	-2.161871
52	1	0	1.879623	-3.621028	-0.816601
53	1	0	-0.507035	-3.450198	-1.055932
54	1	0	4.110334	1.253658	-0.654966
55	1	0	4.360980	1.513352	1.066422
56	1	0	4.322151	-0.904569	1.493492
57	1	0	-3.328128	-1.571437	1.433643
58	1	0	-1.774290	-0.805668	1.729737
59	1	0	-1.835824	-2.534186	1.376835
60	1	0	-6.124118	0.044931	-1.812211
61	1	0	-2.494031	1.049607	-2.916446
62	1	0	-2.835842	-0.669087	-3.065149

63	1	0	-1.228426	-0.129911	-2.622934
64	1	0	5.309767	-1.160388	-2.057062
65	1	0	3.792345	-0.285605	-2.288102
66	1	0	5.522105	-2.939824	-0.205242
67	1	0	4.092025	-3.728777	-0.866773
68	1	0	4.138139	-3.280147	0.844587
69	1	0	3.881712	-2.010464	-2.646797
70	1	0	-5.047084	2.569964	-0.492891
71	1	0	-6.737051	2.131141	-0.759374
72	1	0	-5.922820	1.903739	-3.315383
73	1	0	-4.713681	0.704789	-3.754946
74	1	0	-4.233549	2.181150	-2.895045
75	1	0	6.340943	-0.872783	2.216401
76	1	0	8.024136	-1.800452	0.487242
77	1	0	-2.432774	5.685195	-0.679639
78	1	0	-3.424853	4.680269	-1.766654
79	1	0	-1.678574	4.736617	-1.992141
80	1	0	10.042378	-0.221313	0.271860
81	1	0	6.927071	1.910854	-0.090548
82	1	0	7.318824	2.919791	1.321003
83	1	0	9.373863	1.576535	1.715882
84	1	0	9.593826	-1.517950	2.161178
85	1	0	8.942668	0.822940	-1.587171
86	1	0	9.509445	3.275453	0.073927
87	8	0	-5.364480	-2.220771	-1.629942

88	8	0	-2.822728	-3.507611	-1.303025
89	8	0	-8.579506	-0.863334	2.406771
90	1	0	-8.765520	-1.234763	3.276142
91	8	0	-7.969678	1.335604	1.186560
92	6	0	-6.628601	0.035623	3.551629
93	6	0	-7.142850	-0.664657	2.291357
94	6	0	-7.062920	0.242710	1.029750
95	1	0	-7.370490	-0.376124	0.174691
96	6	0	-6.485755	-2.029844	2.091616
97	1	0	-5.544708	0.167530	3.521040
98	1	0	-6.861417	-0.566800	4.434961
99	1	0	-7.098835	1.013978	3.664810
100	1	0	-5.402577	-1.945637	2.016775
101	1	0	-6.861913	-2.506982	1.183970
102	1	0	-6.723307	-2.676065	2.942000
103	1	0	-8.749054	0.948712	1.611287

Conformer b-1

Center Number	Atomic Number	Atomic Type	Coordinates (Angstroms)		
			X	Y	Z
1	8	0	6.026306	-0.267169	-0.233608
2	6	0	3.953000	0.882328	0.356940
3	1	0	4.508565	1.532457	1.036394
4	1	0	3.946346	1.367948	-0.623571
5	6	0	4.672160	-0.458968	0.256527
6	1	0	4.750449	-0.895034	1.260764

7	6	0	3.946781	-1.490495	-0.650504
8	6	0	2.483898	-1.642364	-0.095383
9	1	0	2.594928	-2.006435	0.937363
10	6	0	1.745133	-0.302719	0.023100
11	6	0	2.519417	0.700825	0.867536
12	1	0	2.025277	1.673008	0.885120
13	1	0	2.551864	0.344327	1.905382
14	6	0	1.662065	-2.713384	-0.839126
15	1	0	1.693118	-2.538878	-1.921116
16	1	0	2.119692	-3.693450	-0.688938
17	6	0	0.231495	-2.773992	-0.378347
18	1	0	-0.268519	-3.732703	-0.430600
19	6	0	-0.430818	-1.707185	0.079450
20	6	0	0.215348	-0.348925	0.149203
21	6	0	-1.849002	-1.782820	0.638487
22	6	0	-2.665659	-0.476070	0.256251
23	6	0	-2.020202	0.740718	0.948128
24	1	0	-2.431487	0.885859	1.942199
25	6	0	-0.477870	0.640271	1.083007
26	1	0	-0.075046	1.640903	0.941583
27	1	0	-0.250782	0.378380	2.120565
28	6	0	3.978643	-1.033820	-2.122557
29	1	0	3.512322	-1.778689	-2.770701
30	1	0	5.012325	-0.920750	-2.456430
31	1	0	3.463418	-0.085606	-2.280674

32	6	0	4.689185	-2.836979	-0.534208
33	1	0	4.276470	-3.582263	-1.216629
34	1	0	4.627436	-3.236982	0.483131
35	1	0	5.743764	-2.711247	-0.786280
36	6	0	0.888649	0.195231	-1.110512
37	1	0	0.814036	-0.396556	-2.014818
38	1	0	0.835845	1.266795	-1.274459
39	6	0	-2.579804	-0.330280	-1.287550
40	1	0	-1.555810	-0.173841	-1.613530
41	1	0	-3.183072	0.504930	-1.626665
42	1	0	-2.953965	-1.228587	-1.788723
43	6	0	-1.751783	-2.116158	2.165782
44	1	0	-1.305077	-1.303374	2.735364
45	1	0	-1.118637	-2.997349	2.272158
46	1	0	-2.716786	-2.347411	2.614064
47	8	0	-2.379953	1.928964	0.178255
48	6	0	-2.379533	3.113262	0.827689
49	6	0	-2.753807	4.242326	-0.094631
50	1	0	-2.236041	4.148031	-1.049604
51	1	0	-2.512733	5.192407	0.377722
52	1	0	-3.828375	4.205014	-0.292412
53	8	0	-2.126842	3.229785	2.005573
54	6	0	-2.790602	-2.872095	0.152134
55	6	0	-4.236765	-2.319844	0.337344
56	6	0	-4.193049	-0.798526	0.579755

57	8	0	-2.572874	-3.986912	-0.248777
58	8	0	-5.222480	-3.004128	0.267899
59	8	0	-5.072081	-0.237597	-0.405322
60	6	0	-5.709009	0.970338	0.062155
61	1	0	-5.272228	1.822585	-0.465805
62	6	0	-5.415371	1.033085	1.566165
63	1	0	-4.644989	1.771070	1.796679
64	1	0	-6.302989	1.300194	2.140072
65	6	0	-4.938824	-0.387966	1.902530
66	1	0	-5.823090	-1.034384	1.872345
67	6	0	-4.360034	-0.510089	3.312957
68	1	0	-3.411848	0.009725	3.451367
69	1	0	-4.234663	-1.547357	3.623838
70	1	0	-5.076744	-0.060991	4.006018
71	6	0	-7.211137	0.923063	-0.239858
72	1	0	-7.656622	0.084963	0.316819
73	6	0	-7.633348	0.752980	-1.727028
74	6	0	-7.435175	-0.672016	-2.243781
75	1	0	-6.378765	-0.930952	-2.297616
76	1	0	-7.872871	-0.763035	-3.242494
77	1	0	-7.934170	-1.387345	-1.585694
78	6	0	-6.974705	1.787392	-2.642857
79	1	0	-5.906838	1.591043	-2.760436
80	1	0	-7.105905	2.793515	-2.240635
81	1	0	-7.428455	1.748279	-3.637924

82	8	0	-9.061308	1.021993	-1.650875
83	1	0	-9.390573	1.168001	-2.544510
84	8	0	-7.722738	2.160789	0.254963
85	1	0	-8.606281	2.234206	-0.134118
86	6	0	7.402253	2.122279	0.600614
87	1	0	6.567223	2.277265	-0.089294
88	1	0	7.453604	2.975814	1.275267
89	6	0	8.706561	1.978885	-0.193652
90	1	0	9.557642	2.024434	0.490461
91	6	0	8.736292	0.638694	-0.948999
92	1	0	9.737042	0.478578	-1.355617
93	6	0	8.386764	-0.524310	-0.002633
94	1	0	8.307861	-1.450171	-0.574513
95	6	0	7.059723	-0.252760	0.721182
96	1	0	6.888952	-1.008829	1.492239
97	8	0	7.148424	0.974022	1.436474
98	8	0	9.416549	-0.718985	0.962568
99	1	0	9.294667	-0.057750	1.655748
100	8	0	7.852589	0.702299	-2.075230
101	1	0	6.959042	0.501366	-1.754653
102	8	0	8.849479	3.062128	-1.102300
103	1	0	8.335685	2.807901	-1.883025

Conformer b-4

Center	Atomic	Atomic	Coordinates (Angstroms)		
Number	Number	Type	X	Y	Z

1	8	0	6.020066	-0.234360	-0.229031
2	6	0	3.934433	0.867631	0.412904
3	1	0	4.482943	1.487999	1.125056
4	1	0	3.924708	1.401574	-0.542136
5	6	0	4.666253	-0.460256	0.247375
6	1	0	4.746401	-0.945838	1.228645
7	6	0	3.951996	-1.452256	-0.711155
8	6	0	2.491158	-1.646461	-0.163608
9	1	0	2.607768	-2.057551	0.850638
10	6	0	1.738202	-0.321836	0.018013
11	6	0	2.501513	0.648066	0.910518
12	1	0	1.997845	1.613509	0.973194
13	1	0	2.534992	0.243271	1.930553
14	6	0	1.680598	-2.690447	-0.956739
15	1	0	1.707238	-2.464269	-2.029316
16	1	0	2.149450	-3.671289	-0.853817
17	6	0	0.251755	-2.788788	-0.496961
18	1	0	-0.237562	-3.749687	-0.591984
19	6	0	-0.421752	-1.751793	0.010272
20	6	0	0.208926	-0.390341	0.141241
21	6	0	-1.837271	-1.868803	0.568038
22	6	0	-2.670707	-0.557462	0.242911
23	6	0	-2.039344	0.637046	0.984825
24	1	0	-2.453363	0.737070	1.983179
25	6	0	-0.496035	0.548187	1.117915

26	1	0	-0.104508	1.558824	1.023076
27	1	0	-0.267216	0.241544	2.142668
28	6	0	3.976752	-0.920170	-2.157598
29	1	0	3.510714	-1.632115	-2.841873
30	1	0	5.008475	-0.785373	-2.489318
31	1	0	3.456481	0.032525	-2.263425
32	6	0	4.708569	-2.795043	-0.663356
33	1	0	4.304081	-3.508762	-1.383467
34	1	0	4.650770	-3.247482	0.332127
35	1	0	5.761745	-2.644453	-0.907621
36	6	0	0.876255	0.219337	-1.091047
37	1	0	0.808572	-0.329415	-2.022537
38	1	0	0.811819	1.296766	-1.204224
39	6	0	-2.588379	-0.345515	-1.293243
40	1	0	-1.566432	-0.164928	-1.613444
41	1	0	-3.201059	0.496859	-1.595815
42	1	0	-2.954689	-1.225499	-1.831158
43	6	0	-1.733784	-2.264543	2.079624
44	1	0	-1.287084	-1.474858	2.680859
45	1	0	-1.098488	-3.148043	2.148425
46	1	0	-2.697183	-2.514906	2.520725
47	8	0	-2.411264	1.851587	0.263529
48	6	0	-2.433686	3.007469	0.961353
49	6	0	-2.806722	4.168673	0.079193
50	1	0	-2.171056	4.188795	-0.807601

51	1	0	-2.704489	5.096981	0.637026
52	1	0	-3.840179	4.058026	-0.257870
53	8	0	-2.192707	3.078953	2.145313
54	6	0	-2.766482	-2.948420	0.037522
55	6	0	-4.219148	-2.422120	0.246313
56	6	0	-4.194156	-0.912496	0.552688
57	8	0	-2.535127	-4.043060	-0.409126
58	8	0	-5.196710	-3.114588	0.147467
59	8	0	-5.080144	-0.325794	-0.407655
60	6	0	-5.712385	0.873891	0.101701
61	1	0	-5.254159	1.740905	-0.382913
62	6	0	-5.441299	0.861812	1.611212
63	1	0	-4.688598	1.602163	1.889997
64	1	0	-6.341606	1.086430	2.181918
65	6	0	-4.943999	-0.565049	1.890101
66	1	0	-5.819539	-1.221749	1.834874
67	6	0	-4.363718	-0.737125	3.294758
68	1	0	-3.422854	-0.210369	3.456336
69	1	0	-4.223556	-1.784777	3.562244
70	1	0	-5.086914	-0.327602	4.005484
71	6	0	-7.214866	0.836975	-0.253721
72	1	0	-7.612492	-0.134973	0.051635
73	6	0	-7.548857	1.070141	-1.753270
74	6	0	-9.056687	0.896288	-1.977115
75	1	0	-9.349066	-0.147650	-1.835914

76	1	0	-9.320555	1.181363	-3.000051
77	1	0	-9.624769	1.513842	-1.281357
78	6	0	-6.764715	0.190974	-2.728682
79	1	0	-6.940478	-0.869324	-2.532609
80	1	0	-5.694765	0.377740	-2.657941
81	1	0	-7.092502	0.407650	-3.749999
82	8	0	-7.185590	2.461245	-1.952083
83	1	0	-7.574721	2.763568	-2.779986
84	8	0	-7.900484	1.828496	0.514239
85	1	0	-7.792567	2.651334	0.014624
86	6	0	7.359917	2.160170	0.648362
87	1	0	6.527611	2.314069	-0.045099
88	1	0	7.395831	3.004900	1.334996
89	6	0	8.671670	2.044073	-0.138156
90	1	0	9.517131	2.091726	0.552757
91	6	0	8.724293	0.714503	-0.910883
92	1	0	9.730065	0.572567	-1.311831
93	6	0	8.382225	-0.465454	0.016859
94	1	0	8.319738	-1.384616	-0.567718
95	6	0	7.046163	-0.220533	0.733617
96	1	0	6.878793	-0.989383	1.492722
97	8	0	7.115002	0.997289	1.466604
98	8	0	9.406671	-0.659786	0.987851
99	1	0	9.269629	-0.010398	1.689364
100	8	0	7.848501	0.782443	-2.042998

101	1	0	6.955159	0.566504	-1.731862
102	8	0	8.806979	3.140755	-1.031762
103	1	0	8.302846	2.889395	-1.819717

Experimental and calculated UV and ECD spectra

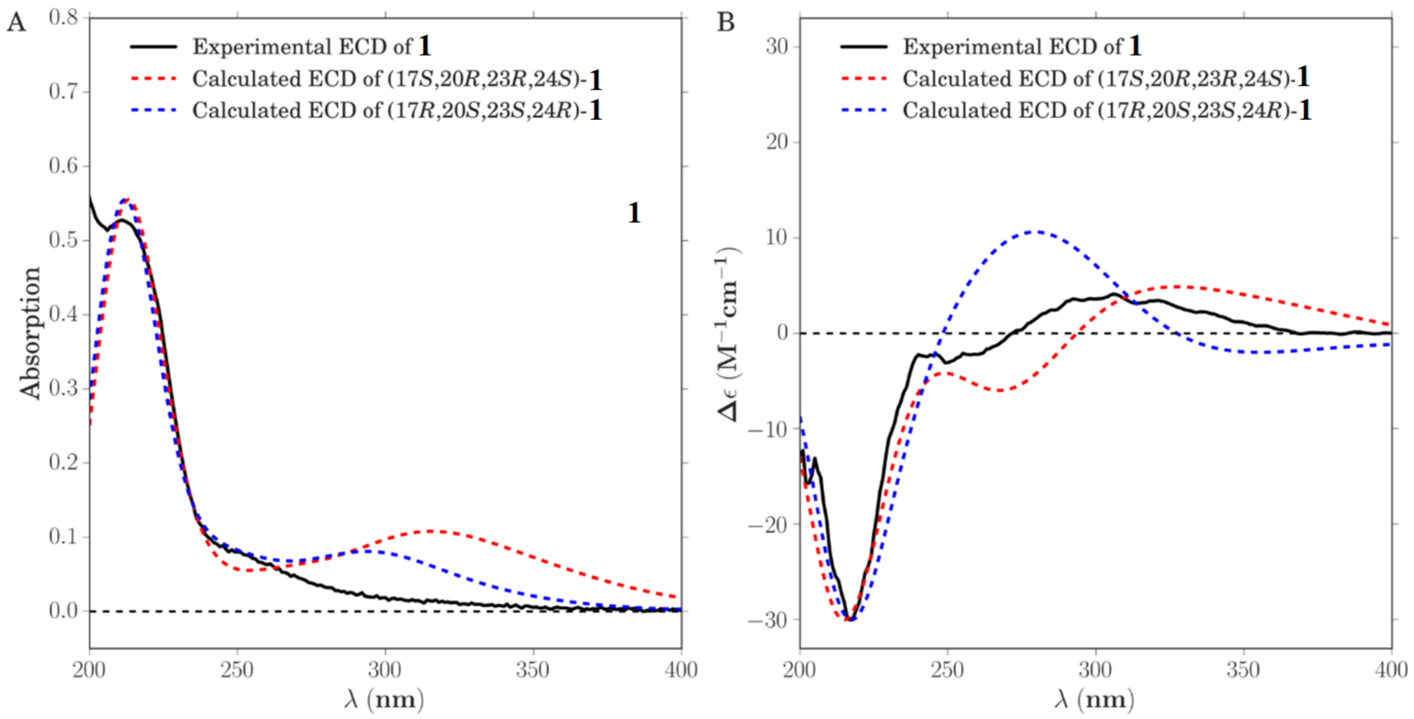

Figure S24. Calculated UV (A) and ECD (B) spectra of configurations **a** (red line) and **b** (blue dash) were compared with the experimental.
